# Selection of carbohydrate-active probiotics from the gut of carnivorous fish fed plant-based diets

**DOI:** 10.1038/s41598-019-42716-7

**Published:** 2019-04-23

**Authors:** Cláudia R. Serra, Eduarda M. Almeida, Inês Guerreiro, Rafaela Santos, Daniel L. Merrifield, Fernando Tavares, Aires Oliva-Teles, Paula Enes

**Affiliations:** 1 CIMAR/CIIMAR - Centro Interdisciplinar de Investigação Marinha e Ambiental, Universidade do Porto, Terminal de Cruzeiros do Porto de Leixões, Avenida General Norton de Matos, s/n, 4450-208 Matosinhos, Portugal; 2 Departamento de Biologia, Faculdade de Ciências, Universidade do Porto, Rua do Campo Alegre s/n, Ed. FC4, 4169-007 Porto, Portugal; 3 CIBIO - Centro de Investigação em Biodiversidade e Recursos Genéticos, InBIO, Laboratório Associado, Universidade do Porto, Campus Agrário de Vairão, 4485-661 Vairão, Portugal; 4 School of Biomedical and Biological Sciences, Plymouth University, 401 Davy Building, Drake Circus, Plymouth, PL4 8AA Devon UK

**Keywords:** Applied microbiology, Marine microbiology

## Abstract

The gastrointestinal microbiota plays a critical role on host health and metabolism. This is particularly important in teleost nutrition, because fish do not possess some of the necessary enzymes to cope with the dietary challenges of aquaculture production. A main difficulty within fish nutrition is its dependence on fish meal, an unsustainable commodity and a source of organic pollutants. The most obvious sustainable alternatives to fish meal are plant feedstuffs, but their nutritive value is limited by the presence of high levels of non-starch polysaccharides (NSP), which are not metabolized by fish. The composition of fish-gut microbial communities have been demonstrated to adapt when the host is fed different ingredients. Thus, we hypothesized that a selective pressure of plant-based diets on fish gut microbiota, could be a beneficial strategy for an enrichment of bacteria with a secretome able to mobilize dietary NSP. By targeting bacterial sporulating isolates with diverse carbohydrase activities from the gut of European sea bass, we have obtained isolates with high probiotic potential. By inferring the adaptive fitness to the fish gut and the amenability to industrial processing, we identified the best two candidates to become industrially valuable probiotics. This potential was confirmed *in vivo*, since one of the select isolates lead to a better growth and feed utilization efficiency in fish fed probiotic-supplemented plant-based diets, thus contributing for sustainable and more cost-effective aquaculture practices.

## Introduction

The gastrointestinal microbial community is a critical actor which impacts host metabolism, immune status and health/disease balance. In the last decade, this relationship has received increased attention particularly in humans, where it is known to control local (at the gut level) health status as well as systemic health^1–3^. The gut microbiota of vertebrates, ranging from mammals to teleost fish, is involved in host appetite control and obesity development^4^, protection against pathogens, immunity enhancement or inflammatory processes^5–7^. Additionally, gut microorganisms respond to a wide range of factors, including dietary composition, and harbor a relevant and diversified enzymatic repertoire that might interfere with host metabolism^8,9^. This is particularly important in fish nutrition, because fish do not possess all of the necessary enzymes to cope with the current aquaculture dietary challenges^10–15^.

Aquaculture output is growing rapidly that has already surpassed fisheries in terms of providing food to meet the growing human population^16^. Fish aquaculture is greatly dependent on fish meal (FM), an unsustainable commodity and a source of organic pollutants, almost exclusively provided by fisheries. This is particularly obvious in carnivorous fish production due to their high dietary protein requirement (40–50%), which is mainly provided by FM. Plant feedstuffs (PF) are sustainable alternatives to FM, and among them, soybean meal (SBM), rapeseed meal (RSM), and sunflower meal (SFM), have been acknowledged as the most promising due to their high protein level, world-wide availability, and reasonable price^17,18^. However, the nutritive value of PF is limited by the presence of several anti-nutritional factors, including high levels of non-starch polysaccharides (NSP) which are not digested by fish^17–20^. NSP content in SBM, RSM, and SFM averages 22–24% and the major NSP components are pectic polysaccharides with arabinose, galactose, and xylose residues predominating^21^. Yet, the proportion of these sugar residues varies between PF with galactose being predominant in SBM, arabinose in RSM, and xylose in SFM^21^. In fish, the carbohydrate-active enzymes (CAZymes) able to hydrolyze the β-glycosidic bonds of NSP are scarce or non-existent^15^. Thus, dietary NSP remain indigestible and cannot be used as energy source. Moreover, indigestible NSP might have detrimental effects on fish performance and nutrient digestibility and on fish health^22^. These adverse effects are associated with the viscous nature of NSP and their interaction with gut epithelium, mucus, and microbiota, which ultimately result on physiological and inflammatory imbalances^22^. Additionally, and contrary to other animal species, such as pigs and poultry, the supplementation of PF based diets with exogenous carbohydrases does not necessarily translate into an effective strategy for improving NSP utilization, as diverging results on their impact on fish growth performance and feed utilization have been reported^23–26^. Therefore, gut microorganisms characterized by a rich secretome are a potential source of *in loco* carbohydrases that may help fish to overcome the mentioned constraints.

Live microorganisms that confer a health benefit to the host when administered in adequate amounts are denominated probiotics^27^. These beneficial microbes might benefit the host, decreasing the incidence of diseases by competing with pathogens for adhesion sites/nutrients; producing natural antimicrobial compounds that inhibit pathogens growth; contributing to a balanced gut microbiota; improving host growth; enhancing host immune system and gastrointestinal histomorphology^28^. Probiotics have also been implicated in bioremediation and water quality improvement by reducing antibiotic usage, contributing to aquaculture sustainability^28^.

Among the bacterial species currently used as probiotics, sporeformers show critical advantages: bacterial spores are remarkably resistant dormant structures^29^, permitting good shelf-storage; spores are easily produced in large scale and can be dehydrated, facilitating feed incorporation. Importantly, spores survive gut transit since they are acid and bile tolerant, and become successfully established in the gut^30^. In particular, *Bacillus subtilis* spores, which enjoy GRAS (Generally Regarded As Safe) status from the U.S. Food and Drug Administration (FDA) and are included in the European Food Safety Authority (EFSA) list of Qualified Presumption of Safety (QPS)^31^, experience exponentially growing applications in biomedicine and biotechnology (as oral vaccines, disinfectants, probiotics or display systems)^32–36^. In fact, different sporeformers are nowadays used as human and animal probiotics^32,34,37,38^, but within European Union just one probiotic has been authorised for use in aquaculture (Bactocell®, LALLEMAND Inc., Canada).

We hypothesize that a selective pressure of diets on fish gut microbiota is a hopeful strategy for an enrichment of bacteria with a secretome able to mobilize dietary components, including the capacity of NSP utilization. Several studies emphasize the presence of carbohydrate-active enzymes in sporeforming species such as *Bacillus* spp.^39^, but few reports exist on the isolation of carbohydrolytic sporeformers from fish gut with probiotic potential that could be administrated in aquafeeds to help fish in their digestive challenges^11,40–42^. Furthermore, available information on carbohydrases-producing bacteria is mainly restricted to amylolytic, cellulolytic and chitinolytic enzymes^41–43^. Thus, screening fish gut microbiota for bacteria capable of producing extracellular digestive enzymes that hydrolyse NSP present in PF (such as mannans, glucans, xylans, arabinans, and galactans), is a promising and unexplored research topic. Gut microbiota isolates showing promising metabolic traits and absence of safety concerns can be used as probiotics in cost-effective and environmental-friendly diets by allowing the host to obtain energy from otherwise indigestible dietary constituents. In fact, native bacteria with probiotic potential will be more apt to become established and persist in the fish gut environment after withdrawal from the diet^44^.

This study describes the isolation, identification and characterization of marine fish gut sporeformers capable of producing carbohydrate-active enzymes that hydrolyse NSP and accesses their potential as probiotics for use in aquafeeds. Sporeformers were isolated from the gut of European sea bass (*Dicentrarchus labrax*) juveniles challenged with PF diets based on SBM, RSM or SFM, which have different NSP profiles. European sea bass was the model species chosen due to its high commercial importance in European aquaculture and its carnivorous feeding habits, thus being more challenging to cope with PF-based diets.

## Results

### Isolation and identification of gut sporeformers

**Figure 1:**
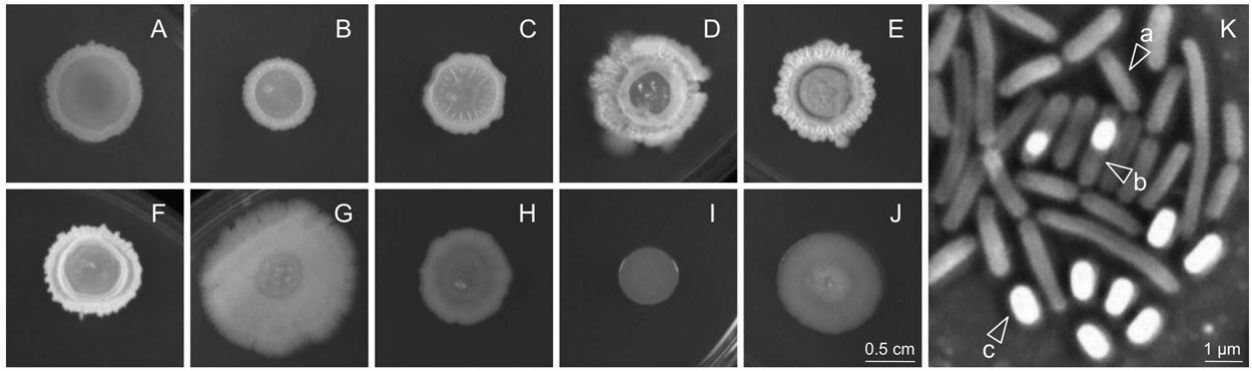
Morphological diversity (Panels A–J) of representative sporeforming fish isolates obtained from European sea bass intestinal contents. Photographs (at the same scale) of colonies grown 24 h in LB (Luria-Bertani) agar medium, are at the same scale defined in Panel J (0.5 cm). Panel K depicts a representative image of the different development stages of sporulation [(a) vegetative cell, (b) sporulating cell (forespore engulfed by the mother cell) and (c) free spore] that were observed in each sporeforming isolate by phase-contrast microscopy. Sporulation was induced by nutrient exhaustion in solid Difco Sporulation Medium (DSM).

**Figure 2:**
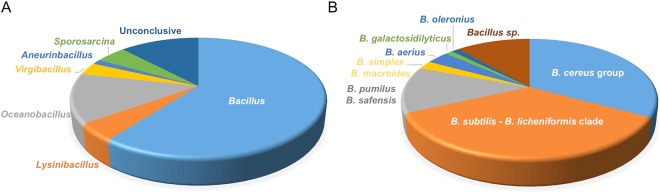
(**A**) Diversity of sporeforming genera obtained from European sea bass digesta samples. (**B**) Distribution of bacterial species within the *Bacillus* genus depicted in panel A.

More than 200 bacterial fish isolates (FI) were obtained from the heat-treated gut contents of European sea bass fed each dietary situation (CTR, SBM, RSM and SFM; Table 1). Following purification, 160 isolates representing different samples and colony morphologies (illustrated in Fig. 1, Panels A to J) were chosen for analysis. Spore production of each isolate, induced by nutrient exhaustion on Difco Sporulation medium, was confirmed by phase-contrast microscopy (Fig. 1, Panel K). All isolates were identified by partially sequencing the 16S rRNA gene revealing a predominance (60%) of *Bacillus* species among European sea bass gut contents (Fig. 2A). *Oceanobacillus* were also present, although to a lower extent (~10%), with the remaining isolates distributed between the genera *Lysinibacillus* and *Sporosarcina* (with 5% each), *Aneurinibacillus* and *Virgibacillus* (with less than 1% of the isolated population, each). Identification to the species level was in most cases inconclusive. Nevertheless, the great majority (>60%) of the isolates belonging to the *Bacillus* genus fall in the *B*. *cereus* group (*B*. *cereus*, *B*. *anthracis*, *B*. *thuringiensis*, *B*. *mycoides*, *B*. *pseudomycoides*, *B*. *weihenstephanensis*, and *B*. *cytotoxicus)* or in the *B*. *subtilis* - *B*. *licheniformis* clade (*B*. *subtilis*, *B*. *vallismortis*, *B*. *mojavensis*, *B*. *atrophaeus*, *B*. *amyloliquefaciens*, *B*. *licheniformis*, *B*. *sonorensis*, *and B*. *tequilensis*) (Fig. 2B).

**Table 1:** Ingredients composition and proximate analysis of experimental diets.

Diets^a^	1st Trial Diets				2^nd^ Trial Diets				
	CTR	SBM	RSM	SFM	CTR−	FI99	FI162	Mix	CTR+
***Ingredients (% dry weight)***									
Fish meal^b^	60.2	38.7	45.2	48.1	5.0	5.0	5.0	5.0	24.5
Fish protein concent^c^	─	─	─	─	5.0	5.0	5.0	5.0	5.0
Pea protein concent^d^	─	─	─	─	4.2	4.2	4.2	4.2	5.0
Soy bean meal^e^	─	30.0	─	─	20.0	20.0	20.0	20.0	10.0
Rapeseed meal^f^	─	─	30.0	─	10.0	10.0	10.0	10.0	─
Sunflower meal^g^	─	─	─	30.0	─	─	─	─	─
Wheat meal^h^	─	─	─	─	8.2	8.2	8.2	8.2	20.0
Wheat gluten^i^	─	─	─	─	10.0	10.0	10.0	10.0	5.0
Corn gluten^j^	─	─	─	─	15.0	15.0	15.0	15.0	10.0
Pregelat. maize starch^k^	23.2	11.6	8.0	4.8	─	─	─	─	─
Fish oil	12.1	13.6	12.4	13.0	14.6	14.6	14.6	14.6	13.0
Bicalcium phosphate^l^	1.0	2.6	1.0	0.6	3.1	3.1	3.1	3.1	3.5
Choline chloride (50%)	0.5	0.5	0.5	0.5	0.5	0.5	0.5	0.5	0.5
Vitamin premix^m^	1.0	1.0	1.0	1.0	1.0	1.0	1.0	1.0	1.0
Mineral premix^n^	1.0	1.0	1.0	1.0	1.0	1.0	1.0	1.0	1.0
Binder°	1.0	1.0	1.0	1.0	1.0	1.0	1.0	1.0	1.0
L-lysine^p^	─	─	─	─	0.6	0.6	0.6	0.6	—
L-methionine^p^	─	─	─	─	0.5	0.5	0.5	0.5	0.4
Taurine^p^	─	─	─	─	0.3	0.3	0.3	0.3	0.2
***Proximate analysis (% dry weight)***									
Dry matter	91.5	92.4	92.7	93.5	91.5	92.0	92.0	91.0	92.6
Crude protein	46.9	46.5	46.3	46.4	44.9	46.5	45.7	46.3	44.9
Crude lipids	17.3	16.1	16.6	16.8	18.1	18.7	18.5	17.8	17.8
Ash	11.3	11.7	11.3	11.1	8.2	8.0	8.0	8.2	8.7

^a^CTR, control fishmeal based diet; SBM, soybean meal based diet; RSM, rapeseed meal based diet; SFM, sunflower meal based diet; CTR**−**, negative control plant-feedstuffs based diet; FI99, FI99 probiotic-enriched diet; FI162, FI162 probiotic-enriched diet; Mix, FI99 + FI162 enriched diet, CTR+, positive control fishmeal based diet. ^b^Steam Dried LT-FM: 1^st^ Trial Diets from Pesquera Diamante, Austral Group, S.A Perú (CP: 74.7% DM; GL: 9.8% DM); 2^nd^ Trial Diets from Copicesa S. A., Spain (CP: 77.1% DM; GL: 11.8% DM). ^c^Fish protein concentrate: Sopropèche G, France (CP: 77.0% DM; GL: 18.4% DM). ^d^Pea protein concentrate: Sorgal, S.A.Ovar, Portugal (CP: 52.5%; CL: 5.9%). ^e^Sorgal, S.A.Ovar, Portugal: 1^st^ Trial Diets (CP: 53.7% DM; GL: 2.1% DM); 2^nd^ Trial Diets (CP: 52.0% DM; GL: 1.9% DM). ^f^Sorgal, S.A.Ovar, Portugal: 1^st^ Trial Diets (CP: 37.5% DM; GL: 4.0% DM); 2^nd^ Trial Diets (CP: 37.3% DM; GL: 5.0% DM). ^g^Sorgal, S.A.Ovar, Portugal (CP: 30.3% DM; GL: 1.0% DM). ^h^Sorgal, S.A. Ovar, Portugal (CP: 14.5% DM; GL: 2.0% DM). ^i^Sorgal, S.A. Ovar, Portugal (CP: 83.1% DM; GL: 1.4% DM). ^j^Sorgal, S.A. Ovar, Portugal (CP: 70.1% DM; GL: 2.8% DM). ^k^Pregelatinized maize starch C-Gel Instant-12016, Cerestar, Mechelen, Belgium. ^l^Premix, Portugal (Calcium: 24%; Total phosphorus: 18%). ^m^Vitamins (mg kg^−1^ diet): retinol acetate, 18,000 (IU kg^−1^ diet); cholecalciferol, 2000 (IU kg^−1^ diet); alfa tocopherol acetate, 35; sodium menadione bisulphate, 10; thiamine-HCl, 15; riboflavin, 25; calcium pantothenate, 50; nicotinic acid, 200; pyridoxine HCl, 5; folic acid, 10; cyanocobalamin, 0.02; biotin, 1.5; ascorbic acid, 50; inositol, 400. ^n^Minerals (mg kg^−1^ diet): cobalt sulphate, 1.91; copper sulphate, 19.6; iron sulphate, 200; sodium fluoride, 2.21; potassium iodide, 078; magnesium oxide, 830; manganese oxide, 26; sodium selenite, 0.66; zinc oxide, 37.5; dibasic calcium phosphate, 8.02 (g kg^−1^ diet); potassium chloride, 1.15 (g kg^−1^ diet); sodium chloride, 0.44 (g kg^−1^ diet). ^j^Aquacube (guar gum, polymethyl carbamide, manioc starch blend, hydrate calcium sulphate) Agil, UK. ^p^ Feed-grade, Sorgal, S.A. Ovar, Portugal.

### Carbohydrolytic activity of gut sporeformers

**Figure 3:**
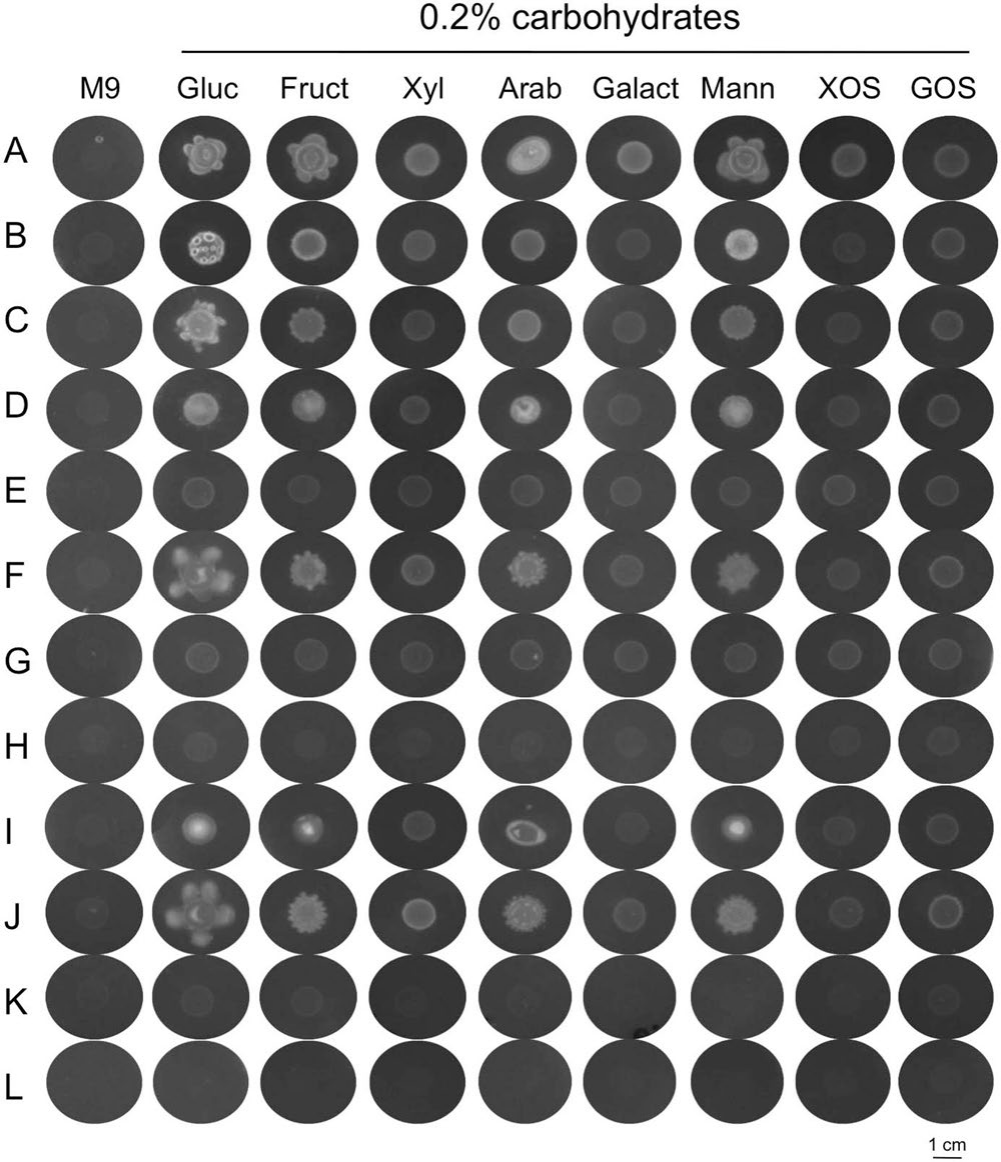
Carbohydrolitic profile of representative sporeformers (**A**–**L**) isolated from the gut of European sea bass, when cultured on solid minimal medium (M9) alone or supplemented with D-glucose (Gluc), D-fructose (Fruct), D-xylose (Xyl), L-arabinose (Arab), D-galactose (Galact), D-mannose (Mann), Xylooligosaccharides (XOS) and Galactooligosaccharides (GOS). All photographs are at the same scale.

The entire collection of 160 isolates was screened for their carbohydrolytic potential by substrate specific culture-based methods, and different profiles of carbohydrate utilization could be assigned to different isolates, as illustrated in Fig. 3. The great majority of isolates grew well on glucose-supplemented medium, but not in the other carbohydrates tested. The quantification of each colony density or volume revealed the 43 isolates with higher and/or broader carbohydrolytic capacity (Supplementary Fig. S1).

### Carbohydrate-active gut sporeformers as putative probiotics for aquaculture

The selected 43 isolates were checked for minimal biosafety requirements to be considered as putative probiotics, following the guidelines from the European Food Safety Authority (EFSA) an the World Health Organization (WHO)^27,45^. The majority (33) of the isolates exhibited some degree of hemolytic activity when cultivated on 5% sheep blood agar plates, with 14 isolates showing strong or β hemolysis (Table 2). Half of the isolates revealed to be resistant to at least 1 antimicrobial, and 10 isolates were resistant to 2 or more antimicrobials, defined as MR in Table 2 and detailed in Supplementary Table S2. These tests allowed selecting a strict group of 11 isolates as good candidates to become a probiotic for European sea bass (Table 2, highlighted in bold lettering), as isolates showing strong hemolytic activity or any antimicrobial resistance to the different classes of antibiotics tested were not further studied.

**Table 2:** Characterization and identification of the 43 isolates with broader carbohydrate-activity.

Isolate^a^	Diet^b^	Spores^c^	Catalase^d^	Hemolysis^e^	AbR^f^	16S rRNA sequence analysis	
						Closest known species^g^	% ID
FI86	CTR	+	+	β	—	*B*. *thuringiensis; B*. *cereus*	100
**FI87**	CTR	+	+	γ	—	*Bacillus sp*.	99.2
**FI89**	CTR	+	+	γ	—	*B*. *subtilis*	98.2
FI91	SFM	+	+	β	—	*B*. *cereus*	100
**FI92**	SFM	+	+	α	—	*B*. *pumilus; B*. *safensis*	100
FI94	SFM	+	+	α	MR	*B*. *licheniformis*	100
FI97	SFM	+	+\−	β	—	*B*. *cereus*	100
FI98	SFM	+	+	β	—	*B*. *simplex; B*. *macroides*	100
**FI99**	SFM	+	+	α	—	*B*. *subtilis; B*. *licheniformis*	100
FI100	SBM	+	+	β	R	*B*. sp	99.2
FI101	SBM	+	+	α	R	*B*. *safensis*	99.8
FI105	RSM	+	+	α	MR	*B*. *licheniformis*	100
FI108	RSM	+	+	α	R	*B*. *pumilus*	99
FI109	RSM	+	+	β	R	*B*. *cereus; B*.*subtilis*	99.6
FI114	RSM	+	+	α	R	*B*. *safensis; B*. *pumilus*	100
FI117	CTR	+	+	β	—	*B*. *subtilis; B*. *mojavensis*	99.2
FI120	CTR	+	+	α	MR	*B*. *licheniformis; B*. *aerius*	100
FI121	CTR	+	+	β	—	*B*. *subtilis*	99.6
**FI123**	SBM	+	+	α	—	*B*. *subtilis; B*. *amyloliquefaciens*	100
FI132	RSM	+	+	α	MR	*B*. *licheniformis*	100
FI135	RSM	+	+	γ	MR	*B*. *licheniformis; B*. *aerius*	86.2
FI136	RSM	+	+	α	MR	*B*. *licheniformis*	100
FI139	RSM	+	+	α	MR	*B*. *licheniformis*	100
FI140	RSM	+	+	α	R	*B*. *pumilus*	99.9
FI141	RSM	+	+	β	—	*B*. *licheniformis*	100
**FI142**	SFM	+	+	α	—	*B*. *subtilis; B*. *amyloliquefaciens*	100
FI143	SFM	+	+	β	R	*B*. *cereus*	100
FI144	SFM	+	+	α	MR	*B*. *licheniformis; B*. *aerius*	100
FI146	SFM	+	+\−	β	R	*B*. *cereus*	100
FI148	CTR	+	+	β	—	*B*. *cytotoxicus*	97.8
FI152	SFM	+	+	γ	MR	*B*. *licheniformis*	100
FI153	SFM	+	+	α	R	*B*. *safensis*	99.5
**FI157**	SFM	+	+	α	—	*Bacillus sp*.	100
FI161	SBM	+	+	γ	MR	*B*. *licheniformis*	100
**FI162**	SBM	+	+	α	—	*B*. *subtilis; B*. *licheniformis*	100
**FI164**	SBM	+	+	γ	—	*B*. *simplex; B*. *macroides*	100
FI165	SBM	+	+	β	—	*B*. *subtilis*	76.1
**FI187**	SFM	+	+	γ	—	*Bacillus sp*.	99.5
FI191	SFM	+	+	γ	R	*Bacillus sp*.	100
**FI226**	RSM	+	+	α	—	*Bacillus sp*.	98.7
FI242	SFM	+	+	γ	R	*B*. *licheniformis*	100
FI268	SBM	+	+	γ	R	*B*. *licheniformis*	100
FI276	RSM	+	+	β	—	*B*. *thuringiensis; B*.*cereus*	100

^a^In underlined lettering are the isolates showing strong hemolytic activity or any antimicrobial resistance, discarded from the rest of the study and in bold the 11 isolates used in subsequent tests. ^b^CTR, control fishmeal based diet; SBM, soybean meal based diet; RSM, rapeseed meal based diet; SFM, sunflower meal based diet. ^c^Spores detected by phase-contrast microscopy of 24 h cultures in DSM agar. ^d^Catalase activity tested by resuspending a colony in a 3% solution of hydrogen peroxide (Sigma). ^e^Hemolysis determined on Columbia 5% sheep blood agar plates after incubation at 37 °C for 24, 48 and 72 h (shown is the final reading at 72 h incubation). β-hemolysis, the bacterial hemolytic enzymes completely break down the blood cells; α-hemolysis, the bacterial hemolytic enzymes only partially break down the blood cells; γ-hemolysis corresponds to essentially no hemolytic activity detected. ^f^AbR-Antimicrobial resistance determined by the E-test method against several antibiotics (Table S2). R-resistance to one antimicrobial; MR-resistance to 2 or more antimicrobials; - no resistance phenotype detected. ^g^ Closest known species found using RDP based on partial sequences (600 to 800 nt) of the 16S rRNA gene.

**Figure 4:**
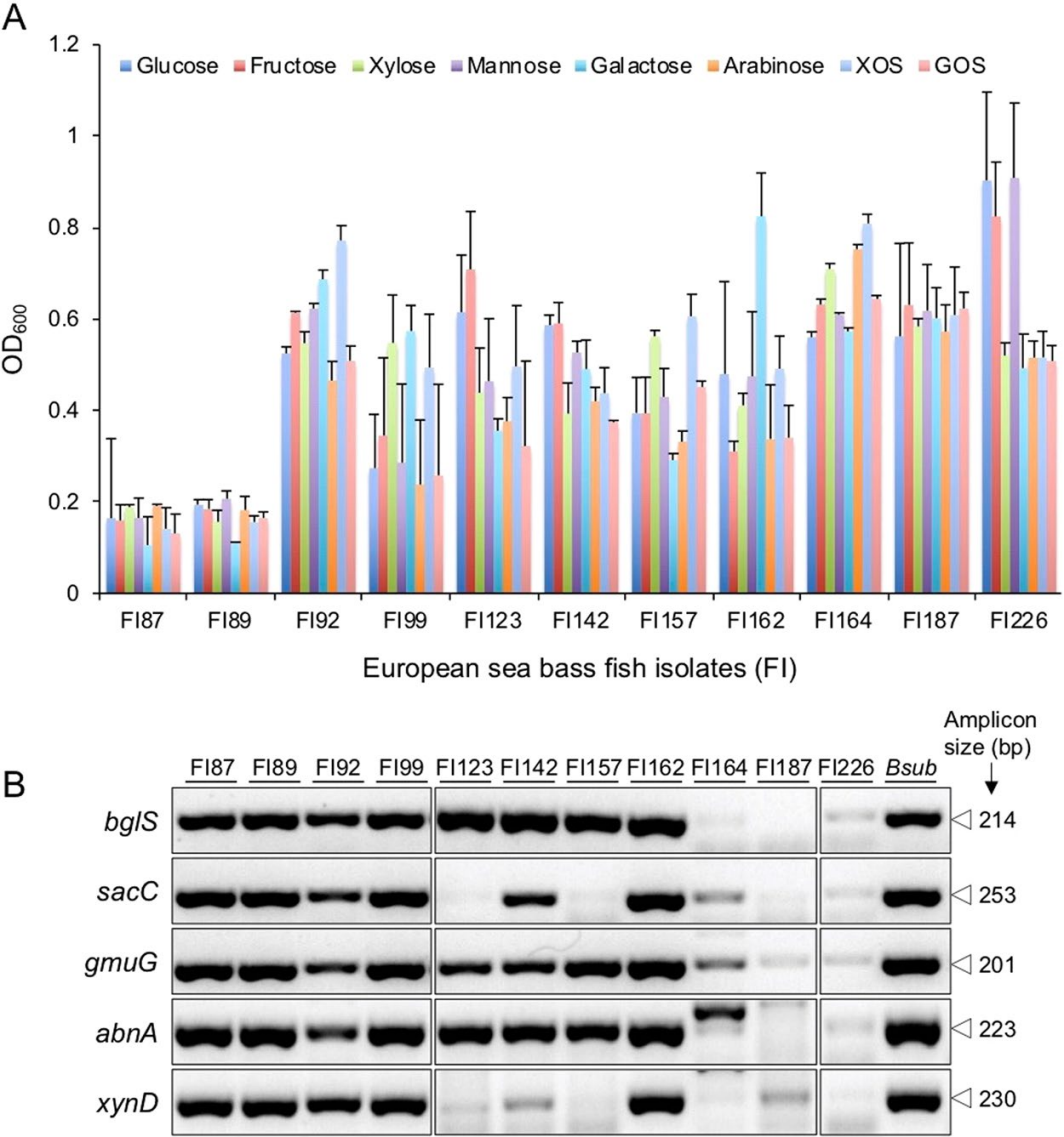
(**A**) Carbohydrolitic profile of the best 11 sporeformers (codes in the x axis) isolated from the intestines of European sea bass, when cultured in liquid minimal medium supplemented with D-glucose, D-fructose, D-xylose, L-arabinose, D-galactose, D-mannose, Xylooligosaccharides (XOS) and Galactooligosaccharides (GOS) for 24 h at 37 °C with agitation. Growth was quantified by measuring the optical density (OD) at an absorbance of 600 nm. The results presented are the average of three independent experiments with error bars representing the standard deviation. (**B**) PCR detection of genes coding for β-glucanase (*bglS)*, levanase or β-D-fructofuranosidase (*sacC)*, mannan endo-1,4-β-mannosidase (*gmuG)*, endo-1,5-α-L-arabinanase (*abnA)* and arabinoxylan arabinofuranohydrolase (*xynD)* carbohydrases in the genome of fish isolates (FI numbers on top of the figure). The amplicon size, in base pairs (bp) is depicted on the right. Figure was constructed using parts of different gels. Corresponding full-length gels are depicted in Supplementary Fig. S2.

The selected 11 isolates were then simultaneously cultured in M9 liquid medium to quantify bacteria growth after 24 h in liquid M9 supplemented with the different carbohydrates (Fig. 4A). The results from 3 independent experiments (Fig. 4A) allowed to eliminate fish isolates FI87 and FI89 from the follow-up tests, after revealing the lowest capacity to metabolize the carbohydrates tested.

The presence of specific carbohydrases coding genes in these 11 isolates was investigated by using oligonucleotide primers specifically designed to target the genes coding for β-glucanase (*bglS)*, levanase or β-D-fructofuranosidase (*sacC)*, mannan endo-1,4-β-mannosidase (*gmuG)*, endo-1,5-α-L-arabinanase (*abnA)*, and arabinoxylan arabinofuranohydrolase (*xynD)* (Table S1). Their broad carbohydrolytic phenotype could not be correlated with the presence of the target genes, since no PCR amplification was obtained for the most promising isolates (FI187 and FI226) while all target genes seem to be present in the worst fish isolates FI87 and FI89 (Fig. 4B).

**Figure 5:**
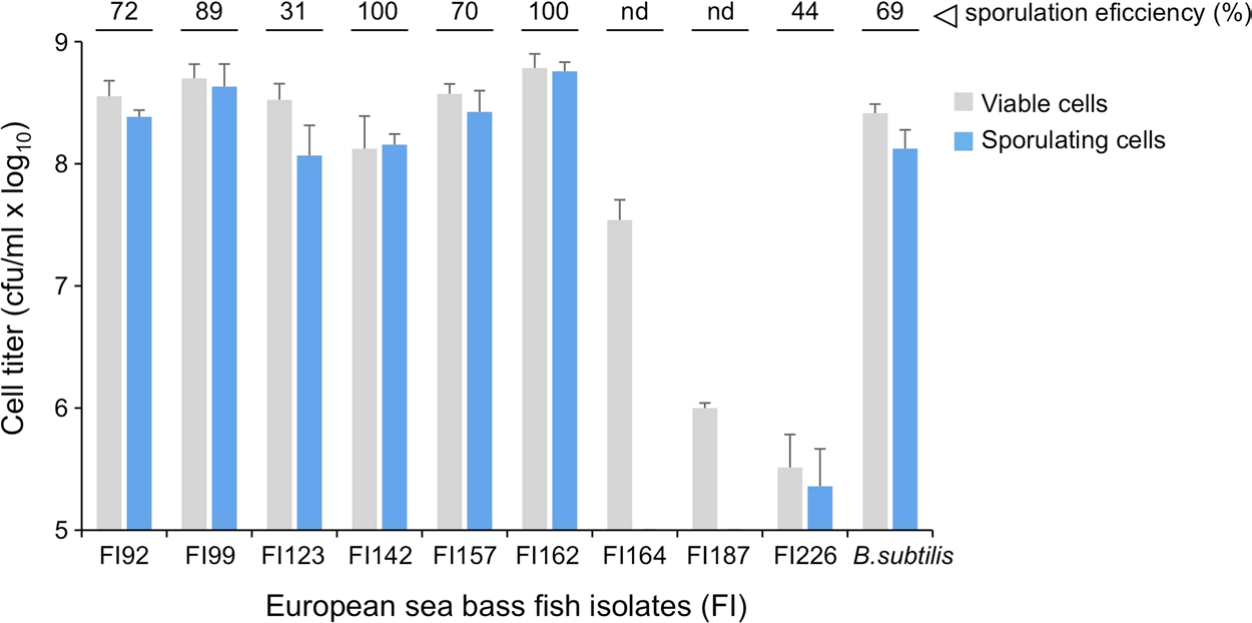
Titer of viable cells present in 24 h DSM cultures of each sporeformer fish isolate (codes in x axis) before (grey, total cells) and after (blue, sporulating or heat resistant cells) a 20 min heat treatment at 80 °C. Sporulation was induced by nutrient exhaustion in liquid Difco Sporulation Medium (DSM) at 37 °C, 150 rpm. Numbers on top of the panel correspond to the percentages (%) of sporulation calculated as the ratio between sporulating cells and total cells. *Bacillus subtilis* 168 was used as control and the results are the average of three independent experiments with error bars representing the standard deviation.

Sporeforming isolates FI92, FI99, FI123, FI142, FI57, FI162, FI164, FI187, and FI226, that simultaneously met the minimal safety requirements to be eligible as probiotic and were the most efficient isolates in metabolizing the carbohydrates tested, were further characterized to determine their sporulation efficiency, an important characteristic for future industrial production and feed incorporation. By comparison with the well-studied standard strain *B*. *subtilis* 168^46^, isolates FI164, FI187, and FI226 did not reach a minimum titer of 107 ml^−1^ heat-resistant cells, after 24 h sporulation induction by nutrient exhaustion in DSM liquid medium (Fig. 5) and were discarded from the subsequent tests. Furthermore, FI187 and DI226 did not even reach that minimum level of total (viable) cells, revealing to be inadequate for future industrial applications. With the exception of FI123, the remaining six isolates presented an efficiency of sporulation higher than 70%, which anticipates a high suitability for cost-effective spores production (Fig. 5).

**Figure 6:**
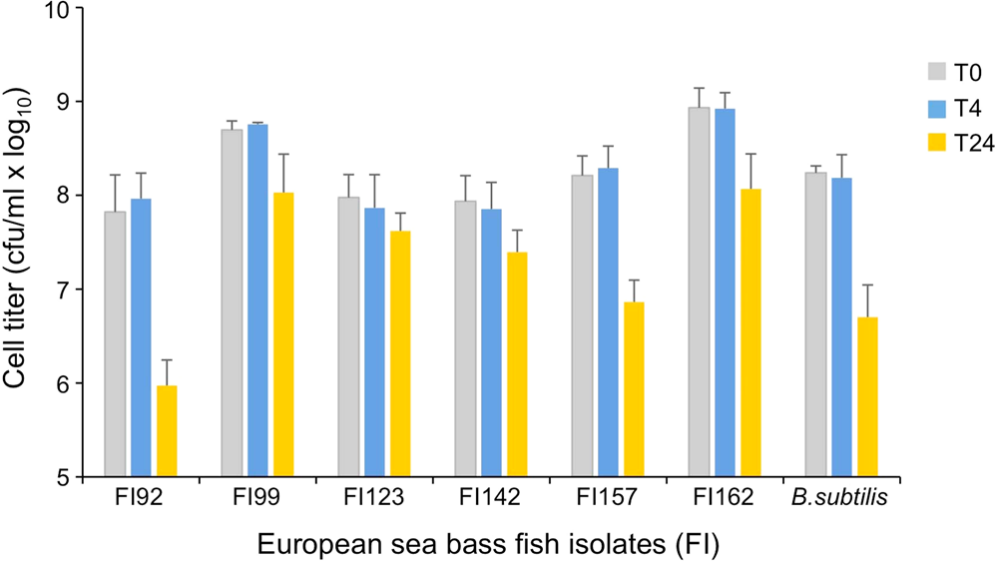
Viability of spores from each sporeformer isolate (codes in x axis) when exposed for 4 h (T4, blue) to simulated stomach conditions (0.85% NaCl, pH 2, containing 3 mg ml^−1^ pepsin) followed by 24 h (T24, yellow) exposition to simulated intestinal condition (LB, pH 8 containing 1 mg ml^−1^ pancreatin and 0.3% bile salts). The initial viable counts (time 0 or T0) are depicted in grey *Bacillus subtilis* 168 was used as control and the results are the average of three independent experiments with error bars representing the standard deviation.

Next, the potential to survive passage through the gastrointestinal tract, important for *in vivo* efficacy, was determined by exposure to sequential simulated stomach and intestinal conditions. Purified spores of isolates FI92, FI99, FI123, FI142, FI57 and FI162 were first subjected during 4 h to acidified NaCl containing pepsin, to mimic stomach conditions, followed by 24 h exposure to alkalinized LB medium containing pancreatin and bile salts. While 4 h in simulated stomach conditions had nearly no effect on the isolates survival, the subsequent 24 h exposure to simulated intestinal conditions lead to a reduction in each bacterial population (Fig. 6). In particular, cell survival was dramatically decreased in isolates FI92 and FI157, similarly to what was observed to the standard strain *B*. *subtilis* 168 (Fig. 6). Isolates FI99 and FI162, which showed higher sporulation efficiency, and consequently higher cell number at time 0, seem to be the best fit to survive in the gut.

**Figure 7:**
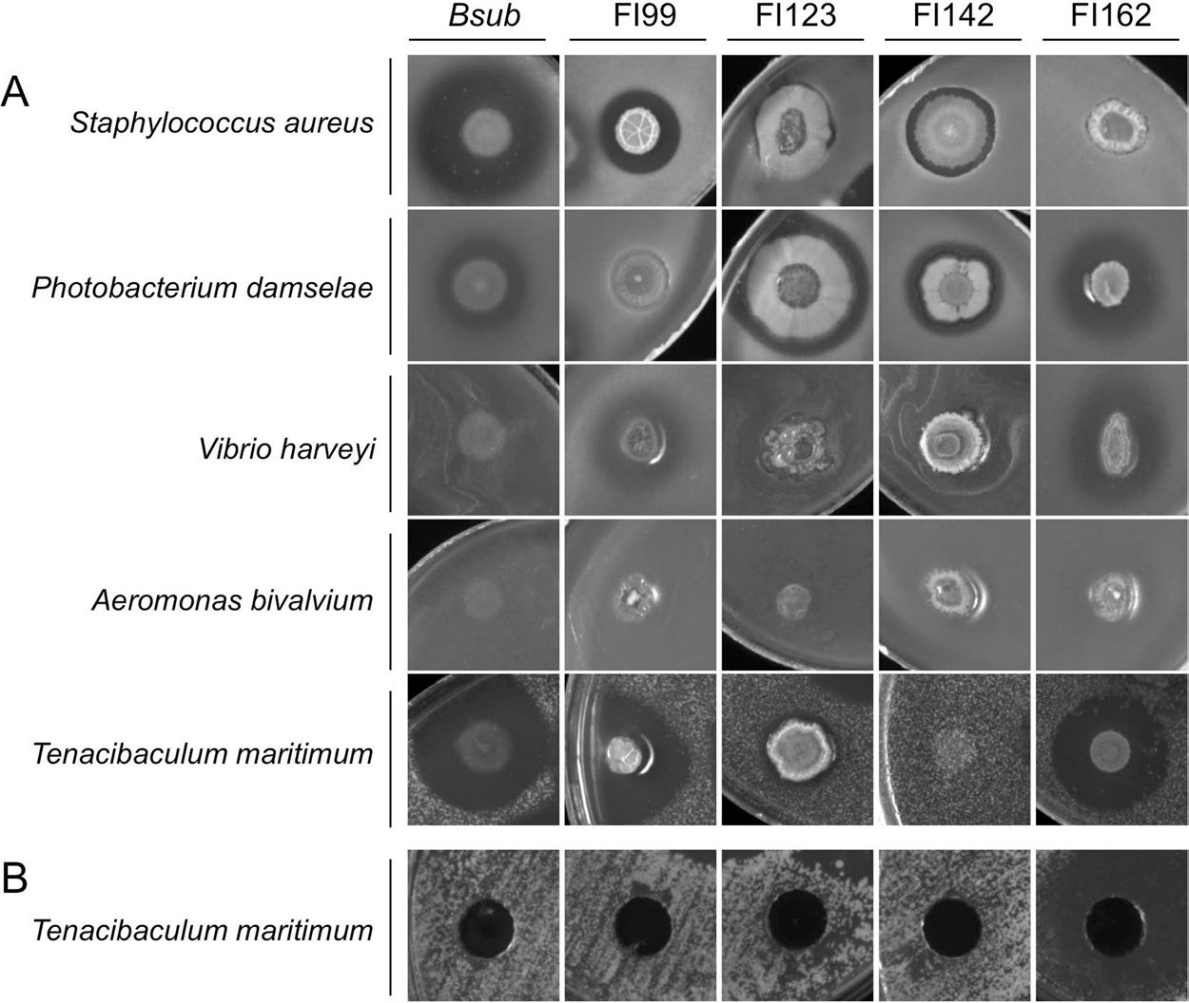
Antimicrobial activity of sporeforming fish isolates FI99, FI123, FI142 and FI162 against different fish pathogens (*Staphylococcus aureus Photobacterium damselae*, *Vibrio harveyi*, *Aeromonas bivalvium* and *Tenacibaculum maritimum*). (**A**) Growth inhibition screened by a colony overlay assay, where the producer strains were inoculated as spots on Luria-Bertani agar plates, grown for 24 h and then covered by Soft Marine Agar (for *Tenacibaculum maritimum)* or Soft Brain Heart Infusion Agar (for all the other) inoculated with indicator pathogenic strains. (**B**) Growth Inhibition screened by a cell-free supernatant assay in which a Marine Agar plate seeded with *Tenacibaculum maritimum* was perforated with 0.5 cm holes and filled with 100 µl of filtered culture medium from overnight grown sporeforming isolates. *Bacillus subtilis* 168 (*Bsub*) was used as control. All photographs are at the same scale.

The remaining four isolates (FI99, FI123, FI142, FI162) were characterized for their antimicrobial activity against several fish pathogenic strains, namely *P*. *damselae*, *V*. *harveyi*, *T*. *maritimum*, *A*. *bivalvium*, and *S*. *aureus*. As illustrated in Fig. 7A, all isolates showed some extent of antimicrobial activity. Strain FI99 was successful in inhibiting the growth of *S*. *aureus*, *T*. *maritimum* and to a lower extent *V*. *harveyi*. FI123 was only active against *Ph*. *damselae*. FI142 inhibited the growth of *S*. *aureus* and of *Ph*. *damselae* while FI162 was active against *Ph*. *damselae*, *V*. *harveyi* and *T*. *maritimum*. The control *B*. *subtilis* 168 could also effectively inhibit the growth of *S*. *aureus*, *Ph*. *damselae* and T. *maritimum*, but this last inhibitory activity was lost when using its cell-free supernatant (Fig. 7B) as opposing to the killing activity observed with the cell-free supernatant of FI162, clearly indicating that this strain produces an extracellular inhibition molecule(s) capable of inhibiting *T*. *maritimum* growth (Fig. 7B).

In an attempt to infer the germination capacity of these strains inside the animal gut, spores of the same four isolates (FI99, FI123, FI142 and FI162) were subject to different germinants, namely L-alanine and a mixture of KCl, glucose, fructose and L-asparagine (AGFK). For the conditions tested, isolates FI123 and FI142 were unable to germinate (Fig. S3), leading to the selection of isolates FI99 and FI162 as the most promising probiotic strains.

A second fish-growth trial assay using challenging plant-based diets (CTR−), revealed that supplementation with 1 × 10^9^ spores g feed^−1^ of FI99 and FI99 + FI162 (Mix) has a positive effect on the final body weight, the weight gain, the feed efficiency and the protein efficiency ratio of European sea bass juveniles, with a tendency to get closer to a FM- based diet (CTR+) (Table 3).

**Table 3:** Growth performance and feed utilization efficiency of European sea bass fed 2^nd^ trial experimental diets^a^.

	Diets				
	CTR−	FI99	FI162	Mix	CTR+
Initial body weight (g)	29.0 ± 0.02	29.0 ± 0.01	29.0 ± 0.03	29.0 ± 0.02	29.0 ± 0.03
Final body weight (g)	74.0 ± 4.8^a^	83.0 ± 1.6^ab^	73.7 ± 6.8^a^	80.0 ± 12.0^ab^	97.0 ± 2.0^b^
Weight gain (%IBW^†^)	155.4 ± 16.6^a^	185.3 ± 5.4^ab^	160.5 ± 23.2^a^	176.0 ± 41.3^ab^	233.8 ± 6.7^b^
Daily growth index^b^	1.73 ± 0.1	1.98 ± 0.0	1.77 ± 0.2	1.89 ± 0.3	2.34 ± 0.1
Feed intake (g kg ABW^−a^ ^§^ day^−b^)	15.4 ± 1.4	16.7 ± 0.3	15.6 ± 0.2	15.8 ± 1.3	17.5 ± 0.1
Feed efficiency^c^	0.82 ± 0.03^a^	0.86 ± 0.02^ab^	0.84 ± 0.03^ab^	0.87 ± 0.06^ab^	0.94 ± 0.01^b^
Protein efficiency ratio^d^	1.82 ± 0.08^a^	1.86 ± 0.05^ab^	1.84 ± 0.07^a^	1.88 ± 0.12^ab^	2.09 ± 0.02^b^

^†^IBW: initial body weight.

^§^ABW: average body weight (initial body weight + final body weight)/2.

^a^Mean values and standard deviation (±SD) are presented for each parameter (n = 3).

Significant differences within the diets are indicated by different letters (Tukey test, P < 0.05).

^b^DGI: ((final body weight^1/3^ - initial body weight^1/3^)/time in days) × 100.

^c^FE: (wet weight gain/dry feed intake).

^d^PER: (wet weight gain/crude protein intake).

Finally, both strains have been deposited in the Spanish culture collection (CECT-Colección Espanola de Cultivos Tipo), under the Budapest Treaty on the International Recognition of the Deposit of Microorganisms for the Purposes of Patent Procedure, and, were submitted for protection with a Provisional Patent Application (PT No. 115101).

## Discussion

The role of gut microbiota in shaping human and animal health is well established, and the potential health benefit of manipulating the gut ecosystem using probiotics is increasingly being accepted^5^. In carnivorous fish such as European sea bass, an ideal probiotic should not only enhance resistance to pathogens (i.e. by competitive exclusion, the most common criteria for selection of probiotic strains) but also help fish in their current dietary challenges, including the utilization of plant-feedstuffs (PF). In this study, the application of a PF-based dietary pressure to modulate European sea bass gut microbiota composition and corresponding metabolic functions revealed to be a successful strategy to find carbohydrate-active bacteria with probiotic potential. In particular, we targeted and isolated spore-forming Bacilli, commonly used in probiotic preparations, mainly due to their extreme resistance characteristics and indefinitely survival, advantageous for industrial applications^29,32,34,38^.

Long viewed as soil inhabitants, *Bacillus* spores are nowadays believed to alternate between the soil/water environment and the gastrointestinal tract as part of their natural life-cycle, being able to germinate, grow and sporulate again inside the animal gut^30,47–49^. Thus it was not surprising that a great variety of sporulating organisms (most rod-shaped but also including the cocci *Sporosarcina* spp.) could be found associated with European sea bass gut samples. By sequencing the 16S rRNA gene, all isolates could be assigned to a genus, being *Bacillus* the most prevalent (>60%). Affiliation to a species based on a single molecular marker (16S rRNA) was limited, as expected^50^. This was the case for isolates belonging to the *B*. *cereus* group (*B*. *cereus*, *B*. *anthracis*, *B*. *thuringiensis*, *B*. *mycoides*, *B*. *pseudomycoides*, *B*. *weihenstephanensis*, and *B*. *cytotoxicus)* or to the *B*. *subtilis* - *B*. *licheniformis* clade (*B*. *subtilis*, *B*. *vallismortis*, *B*. *mojavensis*, *B*. *atrophaeus*, *B*. *amyloliquefaciens*, *B*. *licheniformis*, *B*. *sonorensis*, *and B*. *tequilensis)*, whose 16S rRNA gene sequences obtained do not differ enough to distinguish them^51,52^.

Although several *Bacillus* spp. are quite common in the gut of different animals, including the ones with high-fiber feeding habits, such as soil invertebrates or the giant panda^53,54^, few studies have focused on their carbohydrolytic potential^39^. For example, predominant *B*. *subtilis* strains from the intestinal microbial community of the giant panda, seem to have the capacity to growth in a higher fiber environment^55^, opening the possibility that also in fish, *Bacillus* spp. may have a decisive role in shaping their host digestive capacity towards the efficient utilization of PF-diets. In fact, two recent studies, although limited to cellulase and xylanase activities, reported the isolation of carbohydrate-active *Bacillus* spp. from the gut of different fish species^41,42^. The 160 isolates tested in our study showed different, and in some cases potent, hydrolytic capacities when using as sole carbon source selected carbohydrates including xylose, galactose, arabinose, or mannose. This observation was further sustained by the presence of genes coding for specific extracellular CAZymes that can help fish in obtaining the otherwise unavailable energy trapped in PF. The absence of amplification for these specific genes in some isolates, despite showing broad carbohydrolytic activities, is not surprising considering that some studies suggests that new or substantially different CAZymes involved the metabolization pathways are yet to be found in the Bacilli group of organisms^39,56^. Furthermore, the lack of PCR amplification of these genes, observed with some isolates, may also be caused by mismatches of the primer pairs, due to the difficulty to design gene-specific primers regarding genomic regions poorly conserved.

Probiotic approval within European Union for incorporation into animal feed, including aquafeeds, is subject to strict and exhaustive exigencies following EFSA guidelines on quality, safety, and efficacy of the candidate(s) bacterial strain(s)^57–59^. Besides the obligation of strain deposition in an internationally recognised culture collection, candidate probiotic isolates must be tested for the presence of any acquired antibiotic resistance genes^31,45,60^. Probiotics, which are given to animals in massive amounts, should not contribute to the escalation of antimicrobial resistance by acting as vehicles of transferable genetic determinants. Unfortunately, these rules do not apply worldwide and very recently antimicrobial resistant strains were found in probiotic products used in Vietnamese shrimp culture^61^ or in Chinese human commercial products^62^, with all the risks those findings pose to the aquaculture production sector and to public health^63^. Although EFSA guidelines only require the absence of acquired (transmittable) resistance genes, allowing the use of bacterial strains whose antibiotic resistance is chromosomally encoded, we opted to eliminate from our study all the strains showing any antimicrobial resistance to the different classes of antibiotics tested. Adding to that criterion, strains showing strong hemolytic activity, indicative of virulence potential in several pathogenic bacterial species, including sporeforming ones^64–66^, were also not further tested. These tests allowed to select a group of 11 probiotic candidates that qualify with the minimal biosafety issues to be approved by EFSA.

To demonstrate efficacy, EFSA requires three *in vivo* studies showing statistically-significant effects on each target animal species^59^. To conduct such follow-up studies in European sea bass growth and digestibility trials, it was necessary, for practical reasons, to narrow the group of interesting and potential probiotic candidates. These were subjected to a series of consecutive tests to analyze some desired characteristics on a future probiotic product. First the sporulation yield, an important parameter in industrial and economical terms, was determined by comparison with the well-studied standard laboratory strain *B*. *subtilis* 168^46^. Six isolates demonstrated high yield spore formation, which anticipates a good suitability for cost-effective spores´ production in industrial scale. Additionally, higher sporulation levels might also act as a form of propagation inside the animal gut, maximizing these strains beneficial effect^30,67^. Second, exposure of purified spores to sequential simulated gastric and intestinal conditions, revealed the four isolates best equipped to survive passage through the gastrointestinal tract, important to guarantee their *in vivo* efficacy. In particular, isolates FI99 and FI162, which also showed higher sporulation efficiency, seem to be the best suited to reach, at higher numbers, the intestine where their probiotic action can take place.

To take advantage of these isolates as probiotics, upon passage through the stomach and anterior intestine, spores must germinate to originate new vegetative cells that can produce the enzymes/molecules thought to benefit their host. In nature, spore germination is believed to occur in response to specific nutrients^68,69^. For example *B*. *subtilis* spores are known to germinate in response to L-alanine, L-valine and L-asparagine but not in response to their D-enantiomers^68,69^. Taken this, and although the mechanisms of germination of spores of different Bacilli (independently of their specific species) are thought to be essentially the same, one cannot rule out that some of our isolates might respond efficiently to other germination molecules that might be abundant *in vivo* (inside the animal intestine)^68,69^, explaining the germination failure of isolates FI123 and FI142 under the conditions assayed.

Probiotic candidates FI99 and FI162 were further studied, namely by addressing their *in vivo* efficacy in improving PF diets utilization by European sea bass. This evaluation revealed the potential of isolate FI99 in improving fish performance and feed utilization efficiency, when used as a supplement in plant-feedstuffs-based diets. Fish fed PF-based diet supplemented with FI99 showed better zootechnical results than fish fed the un-supplemented PF-based diet (CTR−), tending to the results of the FM-based diet (CTR+). Future analyses including digestive enzymes activity and gut microbiota modulation, might help explain the results obtained. Also, new growth trials, with increasing levels of both isolates incorporation in the diets, will clarify if fish zootechnical results can get similar to the ones obtained with FM-diet. Furthermore, a comprehensive screening of FI99 and FI162 genomes will potentially allow the identification of new carbohydrases.

Besides their carbohydrolytic potential, these probiotics might also benefit the fish host by minimizing colonization with pathogenic species, known to be especially problematic in marine aquacultures. This is the case of *T*. *maritimum* whose growth was efficiently inhibited *in vitro*, when exposed to both cells and cell-free culture medium of isolate FI162. Probiotic candidates FI99 and FI162 are thus being further studied, namely by addressing their *in vivo* efficacy in improving disease resistance in a *T*. *maritimum* infection model^70^. From the preliminary identification based on the 16S rRNA sequence, these strains are *B*. *subtilis* or *B*. *licheniformis*, both species recognised by EFSA as qualifying for the QPS status^31^ and thus excluding them from the need to perform additional toxicological studies. Nevertheless, sequencing both strains genomes is currently being performed allowing, in a short period, a comprehensive screening of their genomic potential to better meet the EFSA criteria for probiotics. Dissecting these genomes will permit to detail and further document their promising biotechnological value as probiotics and/or as sources of carbohydrases or antimicrobial molecules. For instance, determining the number and type of CAZymes present in each genome will provide deeper understanding on their carbohydrolytic potential but might also allow identification of genomic features responsible for adaptation to life within the gut that may support the role of *Bacillus* spp. as probiotics^39,71–73^. The growing applications of spores in biomedicine and biotechnology (as oral vaccines, probiotics or display systems)^32–35,38,74^, and the fact that there are approximately 30 probiotic strains approved as feed additives in European Union, but only one for aquaculture (Bactocell®, which is not a sporeformer formulation), underscore the importance of this study.

## Materials and Methods

All methods were carried out in accordance with relevant guidelines and regulations, namely in the construction of figures and their compliance with the digital image and integrity policies. All animal experiments were approved by the Animal Welfare Committee of the Interdisciplinary Centre of Marine and Environmental Research (CIIMAR) and carried out in a registered installation (N16091.UDER) and were performed by trained scientists (following FELASA category C recommendations) in full compliance with national rules and following the European Directive 2010/63/EU of the European Parliament and the European Union Council on the protection of animals used for scientific purposes.

### Diets composition

In the first trial, three experimental diets were formulated to be isonitrogenous (47% crude protein), isolipidic (17% crude lipid) and to contain 30% of soy bean meal (SBM diet), 30% of rapeseed meal (RSM diet) or 30% of sunflower meal (SFM diet). A fish meal (FM) based diet was used as the control diet (CTR diet). Fish oil and pregelatinized maize starch were the main lipid and carbohydrate sources, respectively. For the second trial, five experimental diets were formulated to be isoproteic (46%) and isolipidic (18%). A negative control diet (diet CTR**−**), using fish meal (FM) and plant feedstuffs (PF) (soybean meal, rapeseed meal, corn gluten, wheat gluten, and pea protein concentrate) as protein sources at a ratio of 20:80 of protein from FM:PF, respectively. Three other diets were formulated identical to diet CTR**−**, with the incorporation separately of 2 lyophilised pure spores preparations (diet FI99 and diet FI162, respectively) at a dose commonly used in fish diets (1 × 10^9^ spores g feed^−1^), or mixed in equal parts (diet MIX). The fifth diet was a FM-based diet and was used as positive control (diet CTR+). In all diets of the second trial, fish oil was used as the main lipid source.

All diets were supplemented with bicalcium phosphate to avoid phosphorus imbalance. All diet ingredients were finely ground, thoroughly mixed and dry-pelleted in a laboratory pellet mill (California Pellet Mill, CPM Crawfordsville, IN, USA), through a 3.0 mm die for the first trial and a 2.0 mm die in the second trial. Pellets were dried in an oven at 50 °C for 24 h, and then stored at −20 °C in airtight bags until used. Ingredients and proximate composition of all experimental diets are presented in Table 1.

### Animals and experimental conditions

Both animal experiments were performed at CIIMAR, Porto University, Portugal, with European sea bass juveniles obtained from a commercial fish farm (Maresa S.A., Ayamonte, Huelva, Spain). After transportation to the experimental facilities fish were first submitted to a quarantine period of 30 days before transfer to the experimental system where they were allowed to adapt during 15 days. Before the experimental period, fish were fed a commercial diet (Aquasoja Sustainable Feed; Sorgal, Ovar, Portugal) containing 18% lipids and 44% protein. Both trials were conducted in a recirculating aquaculture system (RAS) equipped with 15 fiberglass tanks of 100 L water capacity, thermo-regulated to 22.7 ± 0.8 °C and supplied with a continuous flow of seawater (36.0 ± 0.5 g L^−1^ salinity, circa 7 mg L^−1^ oxygen). The photoperiod was set to 12:12 h light:dark which is provided using artificial illumination. In the first trial, 12 groups of 20 European sea bass with an initial mean body weight of 34.4 g were distributed to each tank and the experimental diets randomly assigned to triplicate groups. The trial lasted 45 days. In the second trial, that lasted 9 weeks, 15 groups of 18 fish with an initial mean body weight of 29.0 g were established and the experimental diets randomly assigned to triplicate tanks. In both trials, fish were fed by hand, twice daily, 6 days a week, until apparent visual satiation. Utmost care was taken to avoid feed losses.

### Sampling

Fish in each tank were bulk-weighed at the beginning and at the end of the trial, after 1 day of feed deprivation. For that purpose, fish were slightly anaesthetized with 0.3 ml l^−1^ ethylene glycol monophenyl ether (Sigma-Aldrich, Steinheim, Germany). On the sampling days (at day 15 after the beginning of the trial and at the end of the trial or day 45), fish were fed several times over the day to guarantee that intestines were full at sampling time. At 4 h after the first meal, 3 fish per tank were randomly sacrificed with an overdose of ethylene glycol monophenyl ether, for collection of biological samples under aseptic conditions. To overcome inter-fish variation the resulting material was pooled into one sample per tank to assess differences between dietary groups. Whole-intestines (without pyloric caeca) were aseptically excised and squeezed to collect the digesta contents.

### Isolation of sporeforming bacteria

Each sample of digesta (1 g) obtained from fish fed the different dietary treatments was homogenized in 9 ml of buffered saline solution (0.9%). Serial dilutions were prepared in Bott & Wilson (B&W) salts and 100 μl aliquots spread on the surface of Luria Bertani (LB) agar medium, after 20 min heat treatment at 65 °C, for sporeformers selection^75^. Plates were incubated at 30 °C in aerobic conditions for up to 5 days. Following selection, sporeformers were isolated and characterized for morphology in Difco Sporulation Medium (DSM), to confirm spore production by phase-contrast microscopy^37,75^. Colonies representing different morphologies were picked at random and purified by restreaking on agar plates of the same media, before storage at −80 °C in LB broth with 30% glycerol. Sporeformers isolates were routinely grown aerobically at 37 °C in LB or DSM. The laboratory strain *B*. *subtilis* 168 (BGSC1A1) was used as a control in most of the experiments described in this study.

### Screening sporeforming bacteria for carbohydrates metabolization

Each sporeformer isolate was cultured on solid M9 minimal medium^76^ = supplemented with 0.2% (w/v) of each of the following carbohydrates: D-glucose (G7528), D-fructose (F3510), D-xylose (X3877), L-arabinose (A3256), D-galactose (G0750), D-mannose (63580), all purchased from Sigma-Aldrich, Steinheim, Germany-Aldrich Co. LLC. The Xylooligosaccharides (XOS) and Galactooligosaccharides (GOS) are commercially available prebiotics from Qingdao FTZ United International Inc. (Quingdao, China) that were added at the same concentration (0.2%). Growth after 24 h at 37 °C was recorded by photographing colonies in a Gel Doc XR System (Bio-Rad) using the Image Lab software v.4.0.1 (Bio-Rad). Growth quantification was assessed by measuring the colony volume on fixed areas with local background subtraction (adjusted volume = [CNT*mm^2^] data counts/mm^2^) using the Quantity One software v.4.6.9 (Bio-Rad). Quantification of carbohydrates utilization in liquid M9 was performed after an overnight enrichment in liquid LB at 37 °C with agitation. Each isolate was diluted to an initial optical density (OD600; absorbance measured at 600 nm) of 0.1 in liquid M9 minimal medium alone or supplemented with 0.2% of the different carbohydrates previously tested. Bacterial growth was followed during 48 h and quantified by measuring the OD600. In both solid and liquid medium assays, results presented were corrected by subtracting the colony volume/OD600 measured in M9 alone.

### Taxonomic identification of putative probiotic isolates

Identification was carried out for all the isolates with promising extracellular carbohydrolytic activities. Total genomic DNA extraction was performed from overnight LB cultures, using the EZNA bacterial DNA purification kit (Omega Bio-Tek, USA), according to the manufacturer’s instructions. PCR amplification of the small-subunit rRNA (16S rRNA) was carried at an annealing temperature of 55 °C using primers 27 F and 1492 R^77^. Each 20 µL reaction contained 1 x DreamTaq Buffer (Thermo Scientific, Vilnius, Lithuania), 0.2 mM of each dNTP (Thermo Scientific, Vilnius, Lithuania), 0.2 μM of each primer (STAB Vida, Lisboa, Portugal), 1 U of DreamTaq DNA Polymerase (Thermo Scientific, Vilnius, Lithuania) and 25 ng of DNA template. The Bioinformatics resources Sequence Match package of the Ribosomal Database Project 11 (http://rdp.cme.msu.edu) and BLAST of the GenBank nonredundant (nr) nucleotide database (http://www.ncbi.nlm.nih.gov) were used to analyse the sequencing data.

### Screening putative probiotic isolates for NSPases

To tentatively obtain a set of primers specific for the genes encoding Non-Starch Polysaccharides degrading enzymes (NSPases), an initial search was conducted at the Protein Knowledgebase - UniProtKB with terms “family:hydrolase AND annotation:(type:location AND secreted) AND taxonomy:“Bacteria [2]”. A file containing bacterial secreted glycosyl hydrolases (GH) was then created and the ones involved in the utilization of NSP of interest were chosen for further analysis. Enzymes chosen included mannanases, mannosidases, arabinofuranosidases, arabinanases, glucosidases, glucanases, fructosidases (fructanases), fructafuranosidases, galactorunases, xylosidases, and xylanases. The protein sequence of each individual enzyme was used to search for similar proteins in the translated nucleotide database (tblastn) (http://www.ncbi.nlm.nih.gov) and to make nucleotide alignments between the sequences obtained with ClustalW algorithm using Geneious R7 v7.1.7 (Biomatters, Auckland, New Zealand). Regions of sequence conservation were chosen to design primer pairs (Supplementary Table S1) with the Vector NTI 10 software (Invitrogen, Carlsbad, CA), with a calculated annealing temperature of approximately 55 °C and an amplicon size of 200 to 250 base pairs (bp). PCR amplification was done essentially as described for the 16S rRNA (previous section), adjusting the annealing temperature to 55 °C and the extension time to 30 s.

### Biosafety issues: antibiotics susceptibility and hemolytic activity

Antimicrobial resistance was studied by testing susceptibility of sporeforming isolates to different classes of antibiotics, namely Macrolides (Erythromycin, EM), Aminoglycosides (Kanamycin, KM, Streptomycin, SM, and Gentamycin, GM), Tetracyclines (Tetracycline, TC), Glycopeptides (Vancomycin, VA) and Cloramphenicol (CL), following the recommendations of the European Food Safety Authority Panel on Additives and Products or Substances used in Animal Feed^45^. Minimal inhibitory concentrations (MIC) were determined using Etest® (bioMérieux, Inc.). Hemolysis was determined on Columbia 5% sheep blood agar plates streaked with colonies from fresh LB plates, after incubation at 37 °C for 24, 48 and 72 h.

### Antimicrobial activity screening assays

The antimicrobial activity of selected sporeforming isolates was assessed by a colony overlay assay, essentially as described in^37^, using as targets different fish pathogens. Zones of growth inhibition around the producer strains spots after 24 h incubation at 25 °C (for *Photobacterium damselae*, *Vibrio harveyi*, *Tenacibaculum maritimum* and *Aeromonas bivalvium*) or 37 °C (for *Staphylococcus aureus*) were considered as positives and the corresponding growth-inhibition halos diameter measured (mm). A cell-free supernatant screening assay was performed by inoculating BHI or Marine Agar (for *T*. *maritimum*) plates with overnight cultures of indicator strains, assuring a uniform and complete coverage of the agar plate. After 15 min rest to allow plates to dry, 1 cm holes where done in the agar and consequently filled with 200 μl of cell-free supernatant of each producer strain, previously centrifuged and filtered through a 0.2 μm cellulose filter, from stationary phase LB cultures (grown overnight at 37 °C). Zones of growth inhibition around the producer strains supernatant holes obtained after 24 h incubation at 25 °C or 37 °C (as before) were considered as positive. All observations were recorded by photographing in a Gel Doc XR System (Bio-Rad) using the Image Lab Software (Bio-Rad).

### Sporulation, germination and resistance to gut environment

The kinetics of spore formation and germination was quantified using adaptations of well-established methods^37,75,76^. Sporulation occurred in DSM for 24 h at 37 °C in an orbital shaker at 200 rpm, and its efficiency was determined by plating serial dilutions made in B&W isotonic buffer (Bott and Wilson salts: 1.24% K_2_HPO_4_, 0.76% H_2_PO_4_, 0.1% trisodium citrate, 0.6% [NH_4_]_2_SO_4_, pH 6.7) on LB agar, before and after a 20 min heat treatment at 80 °C to eliminate vegetative cells. Following 24 h incubation at 37 °C, visible colonies were counted and sporulation efficiency calculated as the titre of colony forming units (CFU mL^−1^) before and after the heat treatment.

Preparation of highly purified spores was done according to a new purification method recently described^78^. In brief, 48 h spores preparations (in liquid DSM) of each isolate were centrifuged for 10 min at 10000 g and 4 °C. Cell pellets were suspended in 50 mM Tris-HCl (pH 7.2) containing 50 µg ml^−1^ of lysozyme, and incubated for 1 h at 37 °C. After a single wash with 1 volume of distilled water (10 min at 10000 g, 4 °C), cell pellets were suspended in 0.05% SDS, followed by three washes with distilled water and finally suspended in 1 volume of distilled water. Spores purity and recovery yields were determined by plating serial dilutions on LB agar, before and after a 20 min heat treatment at 80 °C.

Spore germination in response to the addition of 100 mM L-alanine or to a mixture of 100 mM KCl, 56 mM glucose, 56 mM fructose and 33 mM L-asparagine (AGFK), was performed at 37 °C in 50 mM Tris-HCl, pH 7.5, essentially as previously described^75^.

Potential resistance to gut transit was evaluated by determining the acid and bile tolerance of each selected isolate. For that purpose, 48 h DSM spores preparations were heat-treated for 20 min at 80 °C to eliminate vegetative cells and harvested by centrifugation. After a double wash with Phosphate-buffered saline (PBS), serial dilutions made in B&W salts were plated onto LB agar plates to determine the initial bacterial counts. Spores were then diluted in 1 volume of 0.85% NaCl, pH 2, containing 3 mg ml^−1^ pepsin (Sigma-Aldrich, Steinheim, Germany), to mimic stomach conditions. Following 4 h incubation at 37 °C with agitation, serial dilutions made in B&W were again plated onto LB agar plates to determine bacterial counts, and, after a single wash with PBS, spores were ressuspended in LB, pH 8 containing 1 mg ml^−1^ pancreatin (Sigma-Aldrich, Steinheim, Germany) and 0.3% bile salts (Sigma-Aldrich, Steinheim, Germany). Bacterial incubation continued for 24 h at 37 °C with agitation to mimic passage through the intestine. Finally, serial dilutions made in B&W were again plated onto LB agar plates to determine the final bacterial counts. All plates were incubated at 37 °C during 24 h prior to colonies count.

### Statistical analysis

Data related to growth performance and feed utilization efficiency of European sea bass are presented as mean ± standard deviation. Statistical analysis was conducted by one-way ANOVA using the SPSS 21 software package for Windows (IBM^®^ SPSS^®^ Statistics, New York, USA). Data were tested for normality and homogeneity of variances by the Shapiro-Wilk and Levene’s test, respectively. When normality was not verified, data were transformed prior to ANOVA. Significant differences among groups were determined by the Tukey’s multiple range test. The probability level of 0.05 was used for rejection of the null hypothesis.

## Supplementary information

Supplementary Information

## References

[1] Li D, Wang P, Wang P, Hu X, Chen F (2016). The gut microbiota: A treasure for human health. Biotechnol Adv.

[2] Vernocchi P, Del Chierico F, Putignani L (2016). Gut Microbiota Profiling: Metabolomics Based Approach to Unravel Compounds Affecting Human Health. Front Microbiol.

[3] Schroeder BO, Backhed F (2016). Signals from the gut microbiota to distant organs in physiology and disease. Nat Med.

[4] Fetissov, S. O. Role of the gut microbiota in host appetite control: bacterial growth to animal feeding behaviour. *Nat Rev Endocrinol*, 10.1038/nrendo.2016.150 (2016). 10.1038/nrendo.2016.150 27616451

[5] Montalban-Arques A (2015). Selective Manipulation of the Gut Microbiota Improves Immune Status in Vertebrates. Front Immunol.

[6] Reinoso Webb C, Koboziev I, Furr KL, Grisham MB (2016). Protective and pro-inflammatory roles of intestinal bacteria. Pathophysiology.

[7] Bäumler, A. J. & Sperandio, V. Interactions between the microbiota and pathogenic bacteria in the gut. *Nature* **535**, 10.1038/nature18849 (2016). 10.1038/nature18849 PMC5114849 27383983

[8] Sonnenburg, J. L. & Bäckhed, F. Diet-microbiota interactions as moderators of human metabolism. *Nature* **535**, 10.1038/nature18846 (2016). 10.1038/nature18846 PMC5991619 27383980

[9] Martens EC, Kelly AG, Tauzin AS, Brumer H (2014). The devil lies in the details: how variations in polysaccharide fine-structure impact the physiology and evolution of gut microbes. J Mol Biol.

[10] Gosh K, Roy M, Kar N, Ringo E (2010). Gastrointestinal bacteria in rohu, Labeo rohita: scanning electron microscopy and bacteriological study. Acta Ichthyologica et Piscatoria.

[11] Bairagi A, Ghosh KS, Kumar S, Sen SK, Ray AK (2002). Enzyme producing bacterial flora isolated from fish digestive tracts. Aquaculture International.

[12] Ray AK, Roy T, Mondal S, Ringo E (2010). Identification of gut-associated amylase, cellulase and protease-producing bacteria in three species of Indian major carps. Aquaculture Research.

[13] Lazado CC, Caipang CMA, Kiron V (2012). Enzymes from the gut bacteria of Atlantic cod, Gadus morhua and their influence on intestinal enzyme activity´. Aquaculture Nutrition.

[14] Nayak SK (2010). Role of gastrointestinal microbiota in fish. Aquaculture Research.

[15] Rust, M. B. In *Fish Nutrition*, *3rd Edition* (ed. Halver, J. E., Hardy, R. W.) 367–505 (Academic Press, 2002).

[16] FAO. *The state of world fisheries and aquaculture: Opportunities and challenges*. *Food and agriculture Organization of the United Nations* (2014).

[17] Gatlin DM (2007). Expanding the utilization of sustainable plant products in aquafeeds: a review. Aquaculture Research.

[18] Kaushik, S., Hemre, G. I. In *Improving farmed fish quality and safety* (ed. Lie, Ø.) 300–327 (Woodhead Publishing Ldt, 2008).

[19] Francis G, Makkar HPS, Becker K (2001). Antinutritional factors present in plant-derived alternate fish feed ingredients and their effects in fish. Aquaculture Research.

[20] Krogdahl A, Penn M, Thorsen J, Refstie S, Bakke AM (2010). Important antinutrients in plant feedstuffs for aquaculture: an update on recent findings regarding responses in salmonids. Aquaculture Research.

[21] Knudsen KEB (1997). Carbohydrate and lignin contents of plant materials used in animal feeding. Animal Feed Science and Technology.

[22] Sinha AK, Kumar V, Makkar HPS, De Boeck G, Becker K (2011). Non-starch polysaccharides and their role in fish nutrition – A review. Food Chemistry.

[23] Ai QH (2007). Effects of exogenous enzymes (phytase, non-starch polysaccharide enzyme) in diets on growth, feed utilization, nitrogen and phosphorus excretion of Japanese seabass, Lateolabrax japonicus. Comparative Biochemistry and Physiology.

[24] Ogunkoya AE, Page GI, Adewolu MA, Bureau DP (2006). Dietary incorporation of soybean meal and exogenous enzyme cocktail can affect physical characteristics of faecal material egested by rainbow trout (Oncorhynchus mykiss). Aquaculture.

[25] Farhangi M, Gcarter C (2007). Effect of enzyme supplementation to dehulled lupin-based diets on growth, feed efficiency, nutrient digestibility and carcass composition of rainbow trout, Oncorhynchus mykiss (Walbaum). Aquaculture Research.

[26] Lin S, Mai K, Tan B (2007). Effects of exogenous enzyme supplementation in diets on growth and feed utilization in tilapia, Oreochromis niloticus × O. aureus. Aquaculture Research.

[27] FAO/WHO. (London, Ontario, Canada, 2002).

[28] Verschuere L, Rombaut G, Sorgeloos P, Verstraete W (2000). Probiotic bacteria as biological control agents in aquaculture. Microbiology and Molecular Biology Reviews.

[29] Setlow, P. Spore Resistance Properties. *Microbiol Spectr* **2**, 10.1128/microbiolspec.TBS-0003-2012 (2014). 10.1128/microbiolspec.TBS-0003-2012 26104355

[30] Tam NK (2006). The intestinal life cycle of Bacillus subtilis and close relatives. J Bacteriol.

[31] Efsa-Biohaz. Scientific Opinion on the maintenance of the list of QPS biological agents intentionally added to food and feed (2013 update), European Food Safety Authority Panel on Biological Hazards (EFSA-BIOHAZ). *Efsa Journal* **11** (2013). 10.2903/j.efsa.2010.1944 PMC13085131 42004132

[32] Cutting SM (2011). Bacillus probiotics. Food Microbiol.

[33] Cutting SM, Hong HA, Baccigalupi L, Ricca E (2009). Oral vaccine delivery by recombinant spore probiotics. Int Rev Immunol.

[34] Hong HA, Duc le H, Cutting SM (2005). The use of bacterial spore formers as probiotics. Fems Microbiol Rev.

[35] Potot S, Serra CR, Henriques AO, Schyns G (2010). Display of recombinant proteins on Bacillus subtilis spores, using a coat-associated enzyme as the carrier. Appl Environ Microbiol.

[36] Caselli E (2016). Impact of a Probiotic-Based Cleaning Intervention on the Microbiota Ecosystem of the Hospital Surfaces: Focus on the Resistome Remodulation. PLoS One.

[37] Barbosa TM, Serra CR, La Ragione RM, Woodward MJ, Henriques AO (2005). Screening for Bacillus isolates in the broiler gastrointestinal tract. Appl Environ Microbiol.

[38] Bader J, Albin A, Stahl U (2012). Spore-forming bacteria and their utilisation as probiotics. Benef Microbes.

[39] Manzo N (2011). Carbohydrate-active enzymes from pigmented Bacilli: a genomic approach to assess carbohydrate utilization and degradation. Bmc Microbiol.

[40] Ray AK, Ghosh K, Ringo E (2012). Enzyme-producing bacteria isolated from fish gut: a review. Aquaculture Nutrition.

[41] Das, P., Mandal, S., Khan, A., Manna, S. K. & Ghosh, K. Distribution of extracellular enzyme-producing bacteria in the digestive tracts of 4 brackish water fish species. *Turkish Journal of Zoology* **38** (2014).

[42] Dutta, D. & Ghosh, K. Screening of extracellular enzyme-producing and pathogen inhibitory gut bacteria as putative probiotics in mrigal, Cirrhinus mrigala (Hamilton, 1822). *International Journal of Fisheries and Aquatic Studies* **2** (2016).

[43] Ganguly S, Prasad A (2012). Microflora in fish digestive tract plays significant role in digestion and metabolism. Reviews in Fish Biology and Fisheries.

[44] Balcazar JL (2006). The role of probiotics in aquaculture. Veterinary Microbiology.

[45] Efsa-Feedap. Guidance on the assessment of bacterial susceptibility to antimicrobials of human and veterinary importance, European Food Safety Authority Panel on Additives and Products or Substances used in Animal Feed (EFSA-FEEDAP). *Efsa Journal* **10**, 2740 (2012).

[46] Kunst F (1997). The complete genome sequence of the gram-positive bacterium Bacillus subtilis. Nature.

[47] Hoa TT (2001). Fate and dissemination of Bacillus subtilis spores in a murine model. Appl Environ Microbiol.

[48] Hong HA (2009). Defining the natural habitat of Bacillus spore-formers. Res Microbiol.

[49] Casula G, Cutting SM (2002). Bacillus probiotics: spore germination in the gastrointestinal tract. Appl Environ Microbiol.

[50] Ehling-Schulz M, Messelhausser U (2013). Bacillus “next generation” diagnostics: moving from detection toward subtyping and risk-related strain profiling. Front Microbiol.

[51] Okinaka, R. T. & Keim, P. The Phylogeny of Bacillus cereus sensu lato. *Microbiol Spectr* **4**, 10.1128/microbiolspec.TBS-0012-2012 (2016). 10.1128/microbiolspec.TBS-0012-2012 26999390

[52] Connor N (2010). Ecology of speciation in the genus Bacillus. Appl Environ Microbiol.

[53] Konig H (2006). Bacillus species in the intestine of termites and other soil invertebrates. J Appl Microbiol.

[54] Xue Z (2015). The bamboo-eating giant panda harbors a carnivore-like gut microbiota, with excessive seasonal variations. MBio.

[55] Zhou Z (2015). Transcriptional regulation and adaptation to a high-fiber environment in Bacillus subtilis HH2 isolated from feces of the giant panda. PLoS One.

[56] Rodionov DA (2010). Genomic encyclopedia of sugar utilization pathways in the Shewanella genus. BMC Genomics.

[57] EFSA-NDA. Scientific Opinion on the substantiation of health claims related to non-characterised microorganisms pursuant to Article 13(1) of Regulation (EC) No 1924/2006, European Food Safety Authority Panel on Dietetic Products, Nutrition and Allergies (EFSA-NDA). *Efsa Journal* **7** (2009).

[58] EFSA-NDA. Scientific Opinion on the substantiation of health claims related to non-characterised bacteria and yeasts pursuant to Article 13(1) of Regulation (EC) No 1924/20061, European Food Safety Authority Panel on Dietetic Products, Nutrition and Allergies (EFSA-NDA). *Efsa Journal* **8** (2010).

[59] EFSA-FEEDAP. Guidance for the preparation of dossiers for technological additives, European Food Safety Authority Panel on Additives and Products or Substances used in Animal Feed (EFSA-FEEDAP). *Efsa Journal* **10** (2012).

[60] EFSA-FEEDAP. Guidance on the assessment of the toxigenic potential of Bacillus species used in animal nutrition, European Food Safety Authority Panel on Additives and Products or Substances used in Animal Feed (EFSA-FEEDAP). *Efsa Journal* **12** (2014). 10.2903/j.efsa.2014.3665 PMC13159909 42125372

[61] Noor Uddin GM (2015). Identification and Antimicrobial Resistance of Bacteria Isolated from Probiotic Products Used in Shrimp Culture. Plos One.

[62] Zhu K (2016). Probiotic Bacillus cereus Strains, a Potential Risk for Public Health in China. Front Microbiol.

[63] Cabello, F. C., Godfrey, H. P., Buschmann, A. H. & Dolz, H. J. Aquaculture as yet another environmental gateway to the development and globalisation of antimicrobial resistance. *Lancet Infect Dis*, 10.1016/S1473-3099(16)00100-6 (2016). 10.1016/S1473-3099(16)00100-6 27083976

[64] Popoff MR (2014). Clostridial pore-forming toxins: powerful virulence factors. Anaerobe.

[65] Palma L, Munoz D, Berry C, Murillo J, Caballero P (2014). Bacillus thuringiensis toxins: an overview of their biocidal activity. Toxins (Basel).

[66] Ramarao N, Sanchis V (2013). The pore-forming haemolysins of bacillus cereus: a review. Toxins (Basel).

[67] Serra CR, Earl AM, Barbosa TM, Kolter R, Henriques AO (2014). Sporulation during growth in a gut isolate of Bacillus subtilis. J Bacteriol.

[68] Setlow P (2014). Germination of spores of Bacillus species: what we know and do not know. J Bacteriol.

[69] Paredes-Sabja D, Setlow P, Sarker MR (2011). Germination of spores of Bacillales and Clostridiales species: mechanisms and proteins involved. Trends Microbiol.

[70] Mabrok, M. *et al*. Tenacibaculosis induction in the Senegalese sole (Solea senegalensis) and studies of Tenacibaculum maritimum survival against host mucus and plasma. *J Fish Dis*, 10.1111/jfd.12483 (2016). 10.1111/jfd.12483 27134184

[71] Munir RI (2014). Comparative analysis of carbohydrate active enzymes in Clostridium termitidis CT1112 reveals complex carbohydrate degradation ability. PLoS One.

[72] Lombard V, Golaconda Ramulu H, Drula E, Coutinho PM, Henrissat B (2014). The carbohydrate-active enzymes database (CAZy) in 2013. Nucleic Acids Res.

[73] Park BH, Karpinets TV, Syed MH, Leuze MR, Uberbacher EC (2010). CAZymes Analysis Toolkit (CAT): web service for searching and analyzing carbohydrate-active enzymes in a newly sequenced organism using CAZy database. Glycobiology.

[74] Aps LR (2015). Bacillus subtilis spores as adjuvants for DNA vaccines. Vaccine.

[75] Nicholson, W. L. & Setlow, P. In *Molecular Biological Methods for* Bacillus (eds Harwood, C. R. & Cutting, S. M.) 391–450 (John Wiley & Sons Ltd., 1990).

[76] Harwood, C. R. & Cutting, S. M. In *Molecular biological methods for* Bacillus (eds Harwood, C. R. & Cutting, S. M.) 548 (John Wiley & Sons Ltd, 1990).

[77] Weisburg WG, Barns SM, Pelletier DA, Lane DJ (1991). 16S ribosomal DNA amplification for phylogenetic study. J Bacteriol.

[78] Tavares MB (2013). Bacillus subtilis endospores at high purity and recovery yields: optimization of growth conditions and purification method. Curr Microbiol.

