# Supplementary material for: Selection of carbohydrate-active probiotics from the gut of carnivorous fish fed plant-based diets
Source: Sci Rep. 2019 Apr 23;9:6384. doi: 10.1038/s41598-019-42716-7 (PMC6476879; doi:10.1038/s41598-019-42716-7)
Supplement: Supplementary file 1 — Supplementary Information [file 41598_2019_42716_MOESM1_ESM.pdf]

# Supplementary Information for

## Selection of carbohydrate-active probiotics from the gut of carnivorous fish fed plant-based diets

Cláudia R. Serra, Eduarda M. Almeida, Inês Guerreiro, Rafaela Santos, Daniel L. Merrifield, Fernando Tavares, Aires Oliva Teles, Paula Enes

**Supplementary Table S1.** Oligonucleotide primers used in this study

| Target enzyme[a] / gene[b] | Primer Name | Primer Sequence (5´-3´) |
|---|---|---|
| β-glucanase<br>(GH16 - EC 3.2.1.73) / *bglS* | BglS-339F<br>BglS-553R | AGGGATCGTTTCATCGTTCT<br>TAATAGAGTTTGGCTGCCAATC |
| Levanase (β-D-fructofuranosidase)<br>(GH32 - EC 3.2.1.80) / *sacC* | SacC-106F<br>SacC-336R | CCTCAATATCACTTCACACCGGAG<br>ATCTACAACTGCGCTTCCAGAAAA |
| Mannan endo-1,4-β-mannosidase<br>(GH26-EC 3.2.1.78) / *gmuG* | GmuG-563F<br>GmuG-786R | TCAGGCCGCTGCATGAAATGAACG<br>AATATCCACGTAAGACGCGCCCGG |
| Endo-1,5-α-L-arabinanase<br>(GH43 - EC:3.2.1.99) / *abnA* | AbnA-311F<br>AbnA-564R | GGGCGCCGGACATCCAATACTATA<br>AGTCAGCTTAATGCCGCTCCAAAA |
| Arabinoxylan arabinofuranohydrolase<br>(GH43 - EC:3.2.1.55) / *xynD* | XynD-361F<br>XynD-591R | AAATGGGCAGGTGCGTCATGGGC<br>GTCGTCATCTACAAATACTGCCGG |

[a] the enzyme Glycoside Hydrolase Family (GH) number and the EC number are provide in brackets
[b] gene name in *B. subtilis* strain 168 genome, whose sequence was used to design the oligonucleotide primers

**Supplementary Table S2.** Susceptibility of selected isolates to various antimicrobial agents

| | MIC [a] (µg ml$^{-1}$) | | | | | | |
|---|---|---|---|---|---|---|---|
| Isolate [b] | CL | TC | EM | KM | VA | GM | SM |
| FI86 | 6 | 1.5 | 0.19 | 1.5 | 3 | 0.38 | 1,5-2 |
| FI87 | 4 | 0.094 | 0.094 | 0.75 | 0.75 | 0.125 | 4.-6 |
| FI89 | 4 | 0.125 | 0.094 | 0.75 | 1 | 0.125 | 4 |
| FI91 | 4 | 0.75 | 0.125 | 3 | 2 | 0.75 | 0.75 |
| FI92 | 5 | 0.75 | 0.142 | 0.625 | 0.375 | 0.094 | 0.75 |
| FI94 | > 256 | 4 | > 256 | 3 | 8 | 0.75 | 24 |
| FI97 | 4 | 0.38 | 0.125 | 2 | 2 | 0.28 | 0.38 |
| FI98 | 1.5 | 0.047 | 0.032 | 0.25 | 0.094 | 0.094 | 0,75-1,0 |
| FI99 | 3.5 | 0.22 | 0.0945 | 0.625 | 0.625 | 0.1095 | 1.875 |
| FI100 | 4 | 0.75 | 0.125 | 3 | 8 | 0.75 | 1.5 |
| FI101 | 8 | 0.5 | 0.094 | 1 | 0.25 | 0.38 | 1.5 |
| FI105 | > 256 | 4 | > 256 | 2.-3 | 4 | 0.75 | 16-24 |
| FI108 | 12 | 0.5 | 1 | 1.5 | 0.38 | 0.125 | 1.5 |
| FI109 | 4 | 0.75 | 0.125 | 2 | 4 | 0.38 | 2 |
| FI114 | 12 | 0.38 | 0.5 | 1.5 | 0.5 | 0.19 | 2,-3 |
| FI117 | 6 | 4 | 0.19 | 1 | 1 | 0.064 | 4 |
| FI120 | > 256 | 4,-6 | > 256 | 3 | 4 | 0.5 | 24 |
| FI121 | 6 | 3.-4 | 0.125 | 0.75 | 1 | 0.125 | 4 |
| FI123 | 3.5 | 1.75 | 0.1095 | 0.875 | 0.875 | 0.1095 | 2.5 |
| FI132 | > 256 | 2 | > 256 | 1,5-4 (?) | 6 | 0.75 | 32 |
| FI135 | 14 | 0.315 | > 256 | 1.75 | 2.75 | 0.565 | 3 |
| FI136 | > 256 | 2 | > 256 | 4 | 6 | 0.75 | 32 |
| FI139 | > 256 | 2 | > 256 | 12 | 4 | 0.5 | 19 |
| FI140 | 16 | 0.5 | 0.75 | 1.5 | 0.5 | 0.125 | 3 |
| FI141 | 6 | 1.5 | 0.125 | 0.75 | 1 | 0.125 | 2 |
| FI142 | 3 | 1.5 | 0.125 | 0.875 | 1.125 | 0.172 | 2.5 |
| FI143 | 6 | 0.5 | 0.19 | 2 | 4 | 0.38 | 0,75-2 |
| FI144 | > 256 | 4 | > 256 | 3 | 6 | 0.5 | 16 |
| FI146 | 6 | 0.75 | 0.125 | 2 | 6 | 0.38 | 1.5 |
| FI148 | 3 | 0.094 | 0.125 | 0.75 | 1.5 | 0.25 | 0.5 |
| FI152 | 12 | 0.25 | 0.5 | 2 | 3 | 0.5 | 8 |
| FI153 | 8 | 0.625 | 0.315 | 0.875 | 0.315 | 0.1095 | 0.625 |
| FI157 | 3 | 4.5 | 0.1095 | 0.315 | 1 | 0.079 | 1.75 |
| FI162 | 3.5 | 6 | 0.1095 | 0.75 | 1.125 | 0.094 | 7 |
| FI164 | 5.5 | 20.625 | 2.032 | 0.3125 | 1 | 0.0585 | 4.25 |
| FI165 | 4 | 0.094 | 0.094 | 0.38 | 0.75 | 0.094 | 1 |
| FI187 | 4.5 | 0.1875 | 0.0705 | 0.875 | 0.22 | 0.094 | 2.5 |
| FI188 | 32 | 4.-6 | > 256 | 2 | 2.-3 | 0.38 | 48 |
| FI191 | 8 | 0.047 | 0.064 | 0.38 | 0.125 | 0.016 | 0.75 |
| FI226 | 2 | 0.625 | 0.1095 | 3 | 3 | 0.315 | 0.565 |
| FI242 | 3 | 0.22 | 0.25 | 1.5 | 4 | 0.845 | 2.5 |
| FI268 | 48 | 0.19 | 0.25 | 1.5 | 1.5 | 0.38 | 4 |
| FI276 | 4 | 0.5 | 0.125 | 4 | 3 | 0.38 | 1.5 |

[a] MICs were determined by the Etest® method and in grey boxes are the MIC values above the reference breakpoint (EFSA-FEEDAP, 2012). CL, Cloramphenicol; TC, Tetracyclin, EM, Erythromycin; KM, Kanamycin; VA, Vancomycin; GM, Gentamycin; SM, Streptomycin.

[b] Highlighted in underlined lettering are the isolates showing resistance to 2 or more antimicrobials. All isolates showing any antimicrobial resistance were discarded from the rest of the study.

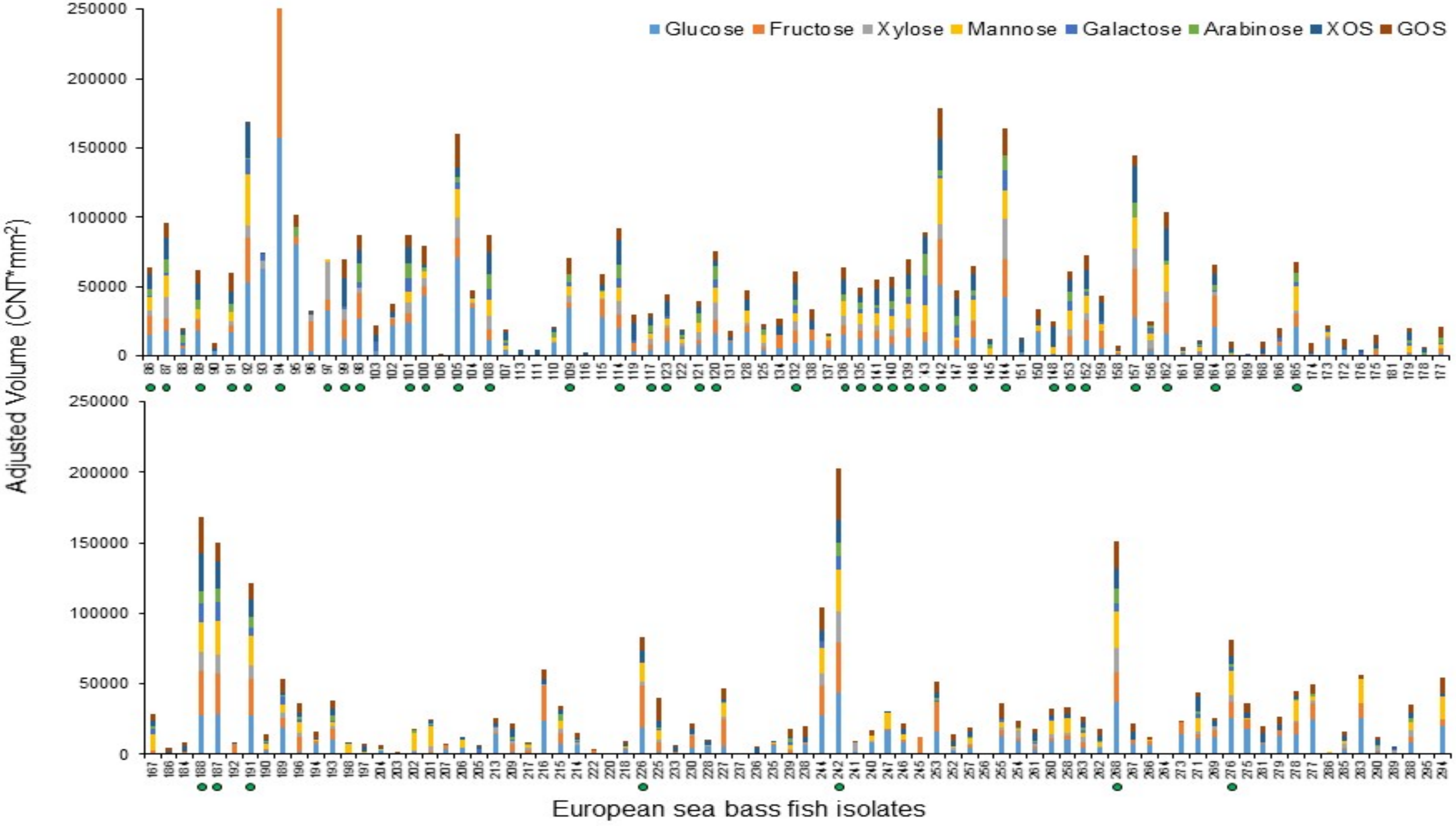


**Supplementary Figure S1.** Carbohydrolitic profile of the 161 sporeformers (number in the x axis) isolated from the gut of European sea bass, when cultured on solid minimal medium supplemented with D-glucose, D-fructose, D-xylose, L-arabinose, D-galactose, D-mannose, Xylooligosaccharides (XOS) and Galactooligosaccharides (GOS). Growth was quantified by measuring the colonies volume from images like the ones presented in Figure 3 using Quantity One software V.4.6.9 (Bio-Rad) with local background subtraction method (adjusted volume=[CNT*mm$^2$] data counts/mm$^2$).

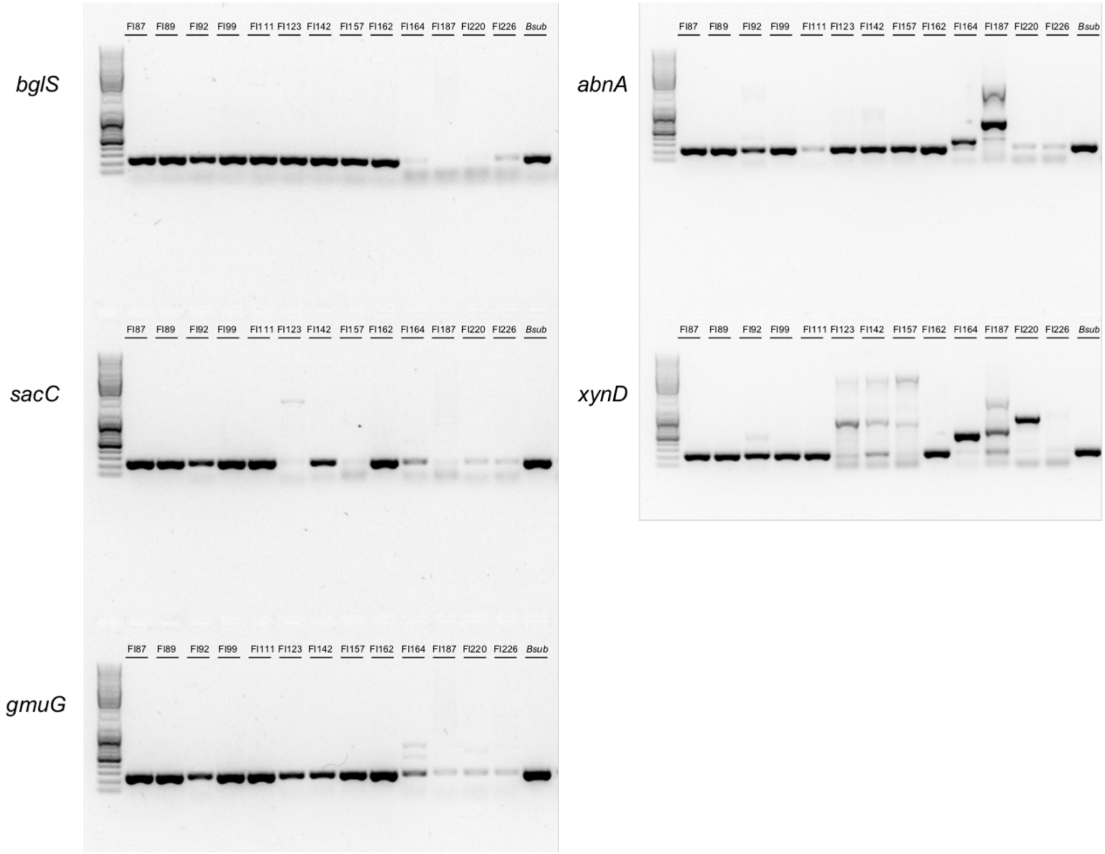


**Supplementary Figure S2.** Full-length agarose gels used for resolution of PCR products from genes coding for β-glucanase (*bglS),* levanase or β-D-fructofuranosidase (*sacC),* mannan endo-1,4-β-mannosidase (*gmuG),* endo-1,5-α-L-arabinanase (*abnA)* and arabinoxylan arabinofuranohydrolase (*xynD)* carbohydrases, in the genome of 13 fish isolates (FI numbers on top of the figure). Parts of gels showing the amplicons of isolates FI87, FI89, FI92, FI99, FI123, FI142, FI157, FI162, FI164, FI187, FI226 and of *B. subtilis* 168 (*Bsub*) were used in the construction of Figure 4.

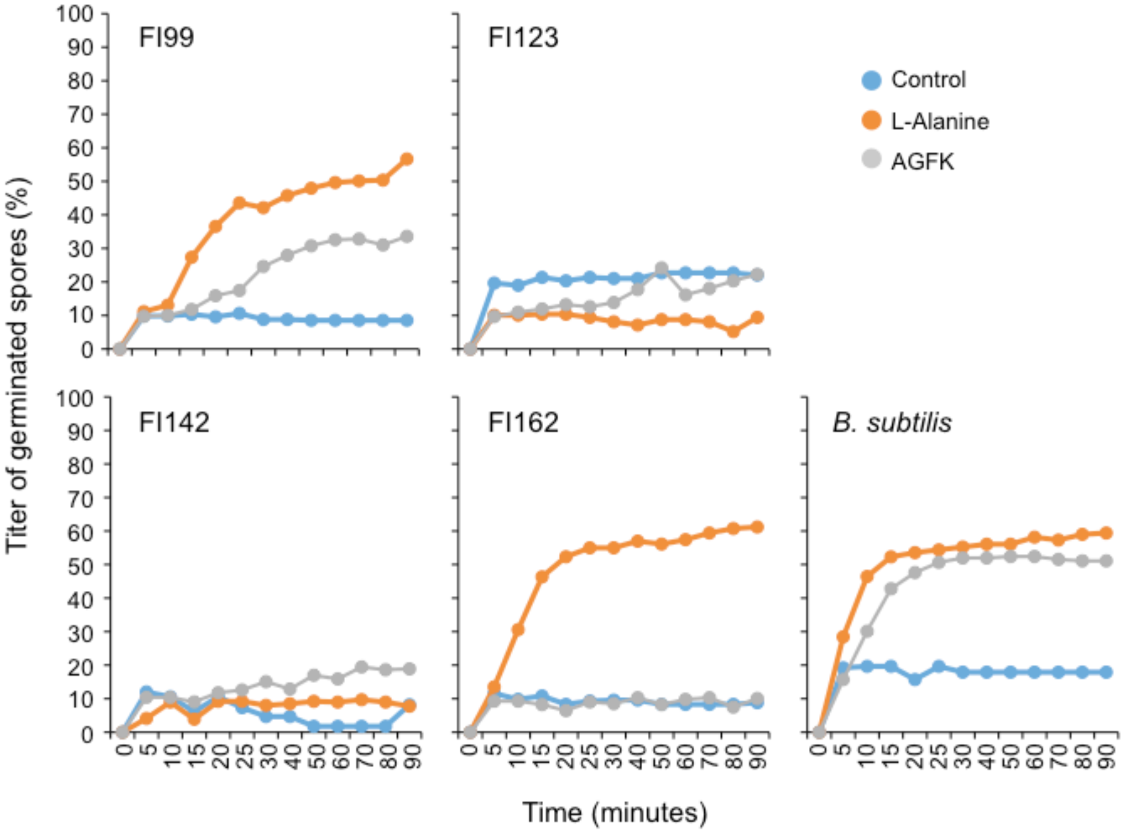


**Supplementary Figure S3.** Germination of populations of purified spores of sporeformers fish isolates FI99, FI123, FI142 and FI162 at 37ºC in 50mM Tris-HCl, pH7.5 (control, blue circles) or in response to the addition of 100mM L-alanine (orange circles) or a mixture of 100mM KCl, 56mM glucose, 56mM fructose and 33mM L-asparagine (AGFK, grey circles). *Bacillus subtilis* 168 was used as control.
